# VvDLK negatively regulates cold tolerance by interacting with Vv14-3-3A in grapevine

**DOI:** 10.1093/plphys/kiag728

**Published:** 2026-09-30

**Authors:** Jiang Wu, Wenjing Shi, Sifang Luo, Lucca Magnus Armlich, Yijing Wu, Qianwei Liu, Xiao Chen, Ruslan Eliseev, Niklas Karsten Kretzler, Sai Kumar Halavath, Pingyin Guan, Peter Nick

**Affiliations:** Molecular Cell Biology, Joseph Gotlieb Kölreuter Institute of Plant Sciences, Karlsruhe Institute of Technology, Fritz-Haber-Weg 4, Karlsruhe 76131, Germany; Molecular Cell Biology, Joseph Gotlieb Kölreuter Institute of Plant Sciences, Karlsruhe Institute of Technology, Fritz-Haber-Weg 4, Karlsruhe 76131, Germany; Department of Pomology, College of Horticulture, China Agricultural University, No. 2 Yuanmingyuan West Road, Beijing 100193, China; Department of Pomology, College of Horticulture, China Agricultural University, No. 2 Yuanmingyuan West Road, Beijing 100193, China; Citrus Germplasm Resource Nursery of Jiangxi Province, Institute of Citrus Science Research of Ganzhou, No.101 Gannan Avenue, Ganzhou 341000, China; Molecular Cell Biology, Joseph Gotlieb Kölreuter Institute of Plant Sciences, Karlsruhe Institute of Technology, Fritz-Haber-Weg 4, Karlsruhe 76131, Germany; Molecular Cell Biology, Joseph Gotlieb Kölreuter Institute of Plant Sciences, Karlsruhe Institute of Technology, Fritz-Haber-Weg 4, Karlsruhe 76131, Germany; Molecular Cell Biology, Joseph Gotlieb Kölreuter Institute of Plant Sciences, Karlsruhe Institute of Technology, Fritz-Haber-Weg 4, Karlsruhe 76131, Germany; Molecular Cell Biology, Joseph Gotlieb Kölreuter Institute of Plant Sciences, Karlsruhe Institute of Technology, Fritz-Haber-Weg 4, Karlsruhe 76131, Germany; Molecular Cell Biology, Joseph Gotlieb Kölreuter Institute of Plant Sciences, Karlsruhe Institute of Technology, Fritz-Haber-Weg 4, Karlsruhe 76131, Germany; Molecular Cell Biology, Joseph Gotlieb Kölreuter Institute of Plant Sciences, Karlsruhe Institute of Technology, Fritz-Haber-Weg 4, Karlsruhe 76131, Germany; Molecular Cell Biology, Joseph Gotlieb Kölreuter Institute of Plant Sciences, Karlsruhe Institute of Technology, Fritz-Haber-Weg 4, Karlsruhe 76131, Germany; Department of Pomology, College of Horticulture, China Agricultural University, No. 2 Yuanmingyuan West Road, Beijing 100193, China; Molecular Cell Biology, Joseph Gotlieb Kölreuter Institute of Plant Sciences, Karlsruhe Institute of Technology, Fritz-Haber-Weg 4, Karlsruhe 76131, Germany

## Abstract

Sudden cold snaps due to blurred seasonality are a rising challenge to agriculture in times of climate change. Swift activation of cold signaling is crucial for resilience. Dual-localization kinesins convey the cold signal from the plasma membrane to the nucleus, where this protein modulates cold-dependent gene expression. Using the grapevine homologue as paradigm, we investigated the functional context of VvDLK. We combined live cell and immunofluorescence imaging with pharmacological interference, physiological analysis, and protein-interaction assays upon stable expression in grapevine cells and tomato, or CRISPR-Cas–mediated inactivation of the tomato homologue. Overexpression of *VvDLK* was associated with earlier and more pronounced cold-induced elimination of microtubules in both tomato plants as heterologous and grapevine cells as homologous hosts, followed by reduced cold tolerance, evident from cellular, physiological, and molecular readouts. Conversely, inactivation of the tomato *VvDLK* homologue *SlDLK* improved cold tolerance. VvDLK interacted specifically with Vv14-3-3A, and a 14-3-3 inhibitor attenuated cold-induced microtubule loss. Overexpression of *Vv14-3-3A* also increased cold susceptibility. In addition, VvDLK and Vv14-3-3A both interacted with VvCBF4, while VvDLK reduced VvCBF4 protein accumulation and altered its intranuclear distribution under cold stress. Together, these findings suggest that VvDLK is associated with cold-induced microtubule elimination and attenuates cold tolerance through a signaling module involving Vv14-3-3A and VvCBF4.

## Introduction

Cold stress, as one of the most critical environmental constraints, limits the capacity of plants to realize their genetic potential by impairing metabolic processes and triggering osmotic, oxidative, and additional secondary stresses (Ding et al 2019). While the public debate about climate change is focused on heat and drought stress, in fact, the progressively blurred seasonality also means challenges by sudden cold snaps. For instance, frost tolerance in grapevine is rapidly disappearing upon bud break (Fuller and Telli 1999). Thus, late frost following precocious sprouting during a warm early spring has tremendous economic consequences. For instance, cold snaps in the springs of 2021 and 2024 annihilated up 80% of yield in the vineyards of the Italian Piedmont, the French Languedoc, or the Upper Rhine region. (Del Zozzo et al 2022; Vautard et al 2023). Such events are expected to become more frequent in the future, motivating research into physiological and molecular mechanisms behind cold tolerance.

A central event in cold signaling is the activation of a transcriptional cascade (for a concise review, see Chinnusamy et al 2007) launched by the accumulation of inducer of CBF expression 1 (ICE1). This protein is continuously turned over under warm temperatures but accumulates in the cold because its proteolytic decay is inhibited by phosphorylation (Ding et al 2015). This accumulation deploys the second tier, the so-called C-repeat binding factors (CBFs) that, in turn, will induce adaptive genes for antifreeze proteins, components of redox homeostasis, or the synthesis of abscisic acid as protective phytohormone (Thomashow 1999). While these downstream events will improve cold tolerance, they come with a growth penalty, which explains why they are not constitutively activated, but only upon need (Lee and Thomashow 2012). Thus, when this need is perceived and transduced efficiently, plants will cope better with the challenges of cold stress. It seems that this efficiency is subject to change.

In temperate regions, many plants can acquire frost tolerance following a prolonged period of chilling (for a classic review on this phenomenon, known as cold acclimation or cold hardening, see Guy 1990). As in many other plants, also in grapevine, cold acclimation is mainly under control of CBF4 (Tillett et al 2012). Swift and strong induction of this transcriptional regulator can be used as predictor of cold hardiness in grapevine (Wang et al 2019). Making use of *Vitis amurensis*, a wild species adapted to the harsh winters in North China, rapidly induced transcription factors such as VaPAT1 (Wang et al 2021) and VaMYB4a (Yu et al 2026) have been identified that might act upstream of CBF4. Post-translational control has emerged as an important regulatory layer, as shown by the E3 ubiquitin ligase VviPUB19, which negatively regulates grape cold tolerance by promoting the degradation of ICE and CBF proteins, and by VvPUB26, which positively regulates grapevine cold tolerance by targeting VvPIF4 for degradation (Wang et al 2025; Zhao et al 2026). Similarly, VaMIEL1 modulates cold tolerance by controlling the stability of VaMYB4a and thereby the CBF–COR pathway (Xie et al 2025). These findings indicate that grapevine cold tolerance is regulated not only by *CBF* gene induction, but also by the stability and turnover of cold-related regulatory proteins.

Because low temperature is a physical factor, it must be translated into an output that can be conveyed biochemically. Such signal translators are termed susceptors and correspond to receptors in the sensing of chemical signals (for review, see Wang et al 2020). The susception of cold is not fully understood. Cortical microtubules contribute to the conversion of early biophysical inputs into signaling outputs (Nick 2013). Their cold-induced elimination is linked to a signaling network involving membrane rigidification, Ca^2+^ influx, oxidative burst, and phospholipase activity (Wang and Nick 2017). This concept has meanwhile been adopted also for other abiotic stress signals (Ren et al 2023; Kumar et al 2023). The physiological significance of microtubule elimination cannot be inferred from the structural change alone, as it is observed both in association with cellular damage and during cold acclimation. In tobacco BY-2 cells, overexpression of *OsTTLL12* accelerated cold-induced elimination of cortical microtubules and was accompanied by increased cell mortality under cold stress (Zhang et al 2022). During cold acclimation in cold-tolerant winter wheat cultivars, early and partial elimination of cortical microtubules is transient and precedes their reorganization into cold-stable arrays (Abdrakhamanova et al 2003). Pharmacological stabilization of microtubules with taxol has also been shown to reduce freezing tolerance in root tips of rye (Kerr and Carter 1990) and mesophyll cells of spinach (Bartolo and Carter 1991). These observations suggest that microtubule elimination should be interpreted in relation to its timing, subsequent microtubule recovery and the cold-tolerance phenotype.

When early disassembly of microtubules is needed for effective perception of cold stress, the role of microtubule-associated proteins (MAPs) shifts into focus. In fact, several MAPs were shown to regulate microtubule reorganization during plant responses to abiotic stress (Zhang et al 2017 [heat stress]; Yu et al 2020 [drought stress]; Dou et al 2021 [ABA]; Yang et al 2022 [salt stress]). In addition to structural MAPs, also microtubule motors can participate in stress signaling. For instance, a kinesin light-chain protein identified in cotton, KLCR1, that interacts with the calcium signal relay protein IQD67, is involved in the response to drought (Li et al 2019). Likewise, kinesin 13A confers salt tolerance in rice (Chen et al 2022). For cold stress, a class-XIV kinesin, dual-localization kinesin (DLK), was found in rice to act as modulator (Xu et al 2018). This kinesin decorates cortical microtubules at normal temperatures, but is partitioned to the nucleus when microtubules disappear under cold stress, which is followed by modulated expression of *CBF4* (Xu et al 2022). To what extent DLK conveys this function in other plants, for instance in woody fruit crops, such as grapevine, has not been addressed so far.

DLK is not the only protein that relocates to the nucleus in response to cold stress. Also 14-3-3 proteins, important scaffolds orchestrating abiotic stress signaling in plants (for review, see Huang et al 2022), show cold-induced nuclear import. Upon phosphorylation through the plasma membrane–associated cold responsive protein kinase 1, they are repartitioned to the nucleus, where they assign, in *Arabidopsis thaliana*, the CBF1 to CBF3 for degradation in the proteasome, which is accompanied by a higher susceptibility to cold stress (Liu et al 2017). Because both DLK and 14-3-3 respond to cold stress by moving from the plasma membrane into the nucleus, and because both are linked to the regulation of CBF-dependent cold responses, we wondered whether this functional similarity is reflected by an interaction between the two proteins.

In this study, we demonstrate that the grapevine kinesin, VvDLK, is a negative regulator of cold tolerance in both tomato as a cold-sensitive heterologous host and grapevine cells as a homologous system. Under cold stress, *VvDLK* overexpression is associated with an earlier and more pronounced elimination of cortical microtubules, together with increased cold susceptibility. Evidence from yeast two-hybrid (Y2H), bimolecular fluorescence complementation, split-luciferase, and co-immunoprecipitation (Co-IP) assays support a physical interaction of VvDLK with Vv14-3-3A. In addition, both VvDLK and Vv14-3-3A interact with VvCBF4, a central regulator of grapevine cold acclimation. VvDLK further reduces VvCBF4 protein accumulation and alters its intranuclear distribution. Together, these findings support a model in which VvDLK modulates cold-related signaling from the plasma membrane to the nucleus through a module involving Vv14-3-3A and VvCBF4.

## Materials and methods

### Plasmid construction

The full-length coding sequence (CDS) of *VvDLK* was polymerase chain reaction (PCR) amplified and recombined into pK7WGF2 and pH7WGR2 using the Gateway cloning system (Thermo Fisher Scientific) using *V. vinifera* cv “Pinot Noir” PN40024 as source, the clone from which the grapevine genome had been drafted. The *VvDLK-*truncated variants were PCR amplified and recombined into pK7WGF2 to dissect domain-specific functions. Two grapevine homologues of Arabidopsis 14-3-3 were identified based on a BLAST search and subsequent structure modeling with AlphaFold3, Vv14-3-3A, and Vv14-3-3B. The CDSs of *VvTuB1*, *Vv14-3-3A*, and *Vv14-3-3B* were PCR amplified and recombined into pK7FWG2 using the same procedure. The following constructs were assembled using the ClonExpress II One Step Cloning Kit (Vazyme). For bimolecular fluorescence complementation (BiFC) assays, the CDS of *VvDLK* was recombined into pSPYNE-35S and pSPYCE-35S, that had been linearized with XbaI and BamHI. The CDSs of *Vv14-3-3A* and *Vv14-3-3B* were cloned into XbaI/BamHI-linearized pSPYCE-35S. The CDS of *VvCBF4* was cloned into XbaI/BamHI-linearized pSPYNE-35S in the same manner. For the split-LUC assay addressing *VvDLK*, the CDS was assembled into a BamHI/SalI-linearized pCAMBIA1300-nLUC and a KpnI/PstI-linearized pCAMBIA1300-cLUC, respectively. The CDSs of *Vv14-3-3A* and *Vv14-3-3B* were assembled into a KpnI/PstI-linearized pCAMBIA1300-cLUC. The *VvCBF4* CDS was cloned into the BamHI/SalI-linearized pCAMBIA1300-nLUC vector. For the Y2H assay, PCR-amplified CDS of *VvDLK* was cloned into pGBKT7 and pGADT7, respectively, making use of the EcoRI/BamHI sites. The *Vv14-3-3A* CDS was assembled into a EcoRI/BamHI-linearized pGADT7 and pGBKT7. The *VvCBF4* CDS was cloned into the EcoRI/BamHI-linearized pGADT7 vector. For the Co-IP assay, the *Vv14-3-3A* CDS was assembled into a EcoRI/BamHI-linearized pCAMBIA35S-4×Myc-MCS-3×FLAG. The *VvDLK* CDS was assembled into a KpnI/XbaI-linearized pBinGFP*2*. The *VvDLK* CDS was assembled into a PstI/BamHI-linearized pCAMBIA3300-Flag. The *VvCBF4* CDS was assembled into a PstI/BamHI-linearized pCAMBIA3300-Myc. For genome editing of the tomato homologue *SlDLK*, the vector pYLCRISPR/Cas9 was used as described in Zeng et al (2018). Details for the oligonucleotide primers used for plasmid construction are listed in Table S1.

### Preparation of plant material for transformation

Suspension cells of *V. rupestris* Scheele (Seibicke 2002) were cultivated in a modified Murashige and Skoog medium supplemented with 3% w/v sucrose, 200 mg/L KH_2_PO_4_, 100 mg/L myo-inositol, 1 mg/L thiamine, and 0.2 mg/L 2,4-D as auxin at pH 5.8. The cells were subcultured at weekly intervals, by transferring 8 mL of mature cell suspension into 30 mL of fresh medium in a 100 mL Erlenmeyer flask. The suspension was cultivated at 25 °C in the dark on a horizontal shaker (KS250 basic; IKA Labortechnik) at 150 rpm. *V. rupestris* calli were maintained at 25 °C in darkness in a growth chamber. Seeds of the tomato cultivar “Micro-Tom” (*Solanum lycopersicum* L.) (Shikata and Ezura 2016) were grown in a greenhouse in the Botanic Garden of the Karlsruhe Institute of Technology under a 16-h light/8-h dark photoperiod and a 25 °C day/22 °C night temperature cycle. The seedlings were used at day 12 for transformation.

### Stable genetic transformation of grapevine cells and tomato plants

The *35S:GFP-VvTuB1*, *35S:GFP-AtTuB6*, *35S:VvDLK-GFP*, and *35S:GFP-Vv14-3-3A* were transferred into *Agrobacterium tumefaciens* LBA4404 cells using the freeze–thaw method (Weigel and Glazebrook 2006). The *A. tumefaciens* strain LBA4404 was grown overnight at 28 °C with shaking (180 rpm). At an optical density at 600 nm (OD_600_) of 0.5 to 0.6, cells were harvested by centrifugation, resuspended in the REM medium, and incubated at room temperature for 1 to 2 h. Calli of *V. rupestris* were immersed in the *Agrobacterium* suspension for 30 min, blotted on sterile filter paper to remove excess inoculum, and placed on COM medium for cocultivation (25 °C, dark, 48 h). After coculture, calli were transferred to SEM medium with 75 mg/L kanamycin or 15 mg/L hygromycin and refreshed every 14 d in the dark. After 2 mo selection, newly formed golden-yellow callus became apparent. Presumptive transformants were confirmed for the presence of GFP and RFP using genomic PCR (for primer details, refer to Table S1), as well as detection of the fluorescent signal. The positively checked calli were picked and subsequently propagated in mass spectrometry murashige and skoog (MS) liquid culture supplemented with the appropriate antibiotics. Details of the media are provided in Table S2.

For the stable transformation of tomato, a leaf-disc protocol (Park et al 2003) was used. In brief, the cotyledons of the tomato seedlings were excised and inoculated with the transgenic *Agrobacterium* cells for 20 min. Subsequently, shoot regeneration was induced under kanamycin selection, and the regenerated plantlets raised further and eventually transferred to soil for flowering and fruit-set. Leaf DNA was extracted from individual seedlings for PCR-based genotyping (again based on detection of the GFP and RFP inserts) and verification of specific fluorescence signals. The plants were further propagated to obtain the T2 generation of independent lines for subsequent experiments.

### Inserts for stable transformation

To visualize microtubules in tomato, the marker *35S:GFP-AtTuB6* was employed, while VvDLK was visualized using a *35S:RFP-VvDLK* construct, generated as described previously. Double transformants of tomato-expressing *GFP-AtTuB6* in the background of *35S:RFP-VvDLK* tomato plants were obtained by crossing the respective single transformants and selecting the progeny. For the suspension cell lines of *V. rupestris*, *35S:VvTuB1-GFP*, *35S:GFP-Vv14-3-3A*, and *35S:GFP-VvDLK* were transformed individually in a stable manner. To see their mutual localization, the GFP-fusion of tubulin was cotransformed with *35S:RFP-VvDLK.*

### Gene expression analysis by real-time quantitative PCR

Total RNA was extracted from the *V. rupestris* cells using the Universal RNA Purification Kit (Roboklon) following the instructions of the manufacturer. For extraction of total RNA from tomato the innuPREP Plant RNA kit (Analytik Jena) was employed, again according to manufacturer protocol. Quantity and quality of the obtained RNA were checked spectrophotometrically (NanoDrop), and, in parallel, by electrophoresis on a 0.8% agarose gel. The extracted messenger RNA was reversely transcribed into complementary DNA (cDNA) with the M-MuLV cDNA Synthesis Kit (New England Biolabs) as described by the manufacturer. Amplification of target genes by real-time quantitative PCR (qPCR) was conducted with 1 µg of cDNA in a CFX96TM real-time PCR cycler (Bio-Rad). Relative expression was calculated according to the 2^−ΔΔCT^ method (Livak and Schmittgen 2001). The details for the oligonucleotide primers are given in Table S1. Biological replicates were independent samples obtained from different tomato plants (separate pots) or independent *V. rupestris* suspension cultures (distinct Erlenmeyer flasks) processed under the same treatment; each sample was extracted for RNA separately. qPCR reactions were run in technical triplicates.

### Transient expression assays in *Nicotiana benthamiana*

To assess the role of different domains for the subcellular localization of VvDLK, a series of truncation constructs were tested upon transient expression in *N. benthamiana* leaves (Sparkes et al 2006): *35S:GFP-DLK* to visualize the localization of the full-length protein, *35S:GFP-DLK*Δ*MT* lacking the motor domain, *35S:GFP-DLK*Δ*CCMT* lacking in addition the coiled-coiled domain, *35S:GFP-DLKCC* just encoding the coiled-coiled domain, *35S:GFP-DLKCCMT* spanning coiled-coiled and motor domain, and *35S:GFP-DLKMT* encoding the motor domain alone. To monitor spatial relationships of DLK with microtubules and other partners, the construct *35S:RFP-DLK* was coexpressed with *35S:GFP-AtTuB6* as a microtubule marker, with *35S:GFP-Vv14-3-3A* as potential binding partner, and with pUBC:mCherry-NLS as nuclear marker. To visualize the localization of Vv14-3-3A protein, the construct *35S:GFP-Vv14-3-3A* was coexpressed with pUBC:mCherry-NLS. To visualize the localization of VvCBF4 protein, the construct *35S:VvCBF4-GFP* was coexpressed with pUBC:mCherry-NLS. All constructs were transformed into *A. tumefaciens* (strain GV3101). Those strains were then co-infiltrated with *A. tumefaciens* carrying the p19 protein of tomato bushy stunt virus, an efficient suppressor of gene silencing (Silhavy et al 2002) into leaves from *N. benthamiana* plants grown for 2 wk according to Sparkes et al (2006). After incubation for 48 h, pieces of the injected leaves were excised and transferred to a liquid half-strength MS medium (Duchefa) for analysis by spinning-disk confocal microscopy (see the following).

### Protein accumulation assay

VvCBF4-Myc, VvDLK-Flag and GFP-Flag were individually introduced into *A. tumefaciens* strain GV3101. Transient expression in *N. benthamiana* leaves was performed as described previously. For coexpression assays, the *Agrobacterium* suspensions carrying the indicated constructs were adjusted to an OD_600_ of 0.6, and the final bacterial density was kept constant among all treatments. After infiltration, plants were maintained under normal growth conditions before being either kept at 25 °C or exposed to 4 °C for 6 h. Infiltrated leaf regions were then harvested for protein extraction and immunoblot analysis. VvCBF4-Myc, VvDLK-Flag and GFP-Flag were detected using anti-Myc (TransGen Biotech), anti-Flag (TransGen Biotech), anti-HA (TransGen Biotech), and anti-GFP (TransGen Biotech) antibodies, respectively. Actin immunoblotting and Coomassie Brilliant Blue staining were used to assess protein loading.

### Quantification of cold-induced damage

The tomato seedlings were raised to the 6-leaf stage and then subjected to cold treatment at 4 °C for specified time intervals. After cold treatment, the appearance of the plants was photographically documented. As readout for cold-induced membrane damage relative electrolyte leakage was measured as described in Blum and Ebercon (1981), and oxidative damage quantified by determining malondialdehyde (MDA) content according to Heath and Packer (1968). Maximum quantum efficiency of photosystem II (Fv/Fm) was measured with a chlorophyll fluorescence imaging system (Mini-PAM II; Heinz Walz) according to the manufacturer's instructions. To visualize and quantify cold-induced oxidative stress, hydrogen peroxide was stained with DAB and superoxide with nitro blue tetrazolium (NBT) as described in Fryer et al (2002). Three biological replicates per treatment were analyzed. A biological replicate consisted of the pooled matured leaves from 3 individual plants. For *V. rupestris*, the plates with the calli were collected at day 7 after subcultivation to record their fresh weight, and then replated and transferred to 4 °C for an additional 10 d. Then, they were photographically imaged and measured again for fresh weight. Again, data represent 3 biological replicates, whereby 1 replicate consisted of 3 independent callus pieces taken from different plates.

### Staining microtubules by immunofluorescence

Microtubules were visualized in the transgenic *V. rupestris* cells by immunofluorescence as described in Qiao et al (2010) using custom made microstaining chambers (Nick et al 2000) to allow for efficient exchange of liquids without losing the cells. In brief, aliquots of 500 µL of cell suspension were fixed in 3.7% freshly prepared paraformaldehyde for 30 min in microtubule-stabilizing buffer (50 mM PIPES, 2 mM EGTA, 2 mM MgSO_4_, 0.1% Triton X-100, pH 6.9), washed 3 times for 5 min in microtubule-stabilizing buffer, digested for 20 min in 1% (w/v) macerozym and 0.2% (w/v) pectolyase (both Duchefa), and blocked with 0.5% (w/v) of bovine serum albumin in phosphate-buffered saline (PBS) for 30 min. Then, the samples were incubated overnight at 4 °C with a monoclonal antibody against detyrosinated α-tubulin (T6199, Sigma-Aldrich; 1:250 in PBS). After washing 3 times for 5 min in PBS, the secondary antibody, a polyclonal anti-mouse IgG conjugated to tetramethylrhodamine (T5393; Sigma-Aldrich; 1:100) was added and the samples were incubated for 1 h at 37 °C. After a final wash for 3 times 5 min in PBS, the samples were mounted and immediately used for microscopic imaging.

### Pharmacological treatments

To assess the role of microtubules for the localization patterns, in some experiments, *V. rupestris* cells or excised sections of the infiltrated *N. benthamiana* leaves were incubated for 30 min with 10 μM of oryzalin (Sigma-Aldrich), a drug that specifically eliminates plant microtubules; 100 nM latrunculin B (LatB) (Sigma-Aldrich), an actin polymerization inhibitor; or 20 μM of taxol (Biomol GmbH), a drug that stabilizes microtubules and suppresses their turnover. As specific inhibitor of 14-3-3 activity, we used BV02 (Sigma-Aldrich). To assess the role of Vv14-3-3s on the microtubular cold response, *V. rupestris* cells were pretreated with 5 µM BV02 for 30 min, and subsequently subjected to 4 °C for 1 h. All compounds were diluted from DMSO stock solutions; the final DMSO concentration did not exceed 0.1%. Accordingly, a 0.1% DMSO solvent control was included throughout.

### Determination of cell mortality

Mortality was determined by the Evans blue dye exclusion test (Gaff and Okong’O-Ogola 1971). Evans blue is membrane impermeable and, therefore, does not stain living cells, but penetrates into dead cells resulting in blue staining. For each sample, aliquots of 200 μL cell suspension were transferred into custom-made microstaining chambers (Nick et al 2000), to remove the medium. After staining for 3 min in 2.5% (w/v) Evans blue, the cells were washed 3 times for 5 min with distilled water. Aliquots of 40 μL stained cells were observed under an AxioImager Z.1 microscope (Zeiss) equipped with an ApoTome microscope slider through the filter sets 38 HE. The samples were imaged at 10-times magnification using the 10 × 10 tile function to ensure systematic sampling. Data represent 3 biologically independent experiments, with more than 500 cells analyzed per replicate.

### Bimolecular fluorescence complementation assay

To test the in vivo interactions of VvDLK with Vv14-3-3s and VvCBF4, as well as that between Vv14-3-3s and VvCBF4, BiFC assays were performed (Walter et al 2004) using the vectors pSPYNE (bearing the N-terminal fragment of YFP, amino acids [aa] 1–155) and pSPYCE (bearing the C-terminal fragment of YFP, aa 156–239) described therein. The pSPYNE-VvDLK, pSPYCE-VvDLK, pSPYCE-Vv14-3-3A, pSPYCE-Vv14-3-3B, and pSPYNE-VvCBF4 vectors were transformed into *A. tumefaciens* strain GV3101, and leaves of *N. benthamiana* were co-infiltrated with equal amounts of *Agrobacterium*-expressing pairs of BiFC constructs, along with *A. tumefaciens* expressing the p19 protein. The bacterial suspensions were injected into tobacco leaves. After 48 h, yellow fluorescent protein (YFP) signals were observed using laser scanning confocal microscopy (Zeiss).

### Split-luciferase assay

To probe for interactions between VvDLK and Vv14-3-3s, VvDLK and VvCBF4, and Vv14-3-3A and VvCBF4 in vivo, a split-luciferase complementation strategy was used (Chen et al 2008). Here, the VvDLK was either fused with the N-terminal (nLUC) or the C-terminal (cLUC) fragment of firefly luciferase, while Vv14-3-3A and Vv14-3-3B were fused with the C-terminal fragment. The VvCBF4 was fused with N-terminal (nLUC) fragment of firefly luciferase. The nLUC-VvDLK, cLUC-VvDLK, cLUC-Vv14-3-3A, cLUC-Vv14-3-3B, and nLUC-VvCBF4 were transformed into *A. tumefaciens* strain GV3101. Those strains were then co-infiltrated with *A. tumefaciens* expressing the p19 protein into leaves of *N. benthamiana* for transient expression. The infiltrated plants were kept in continuous light or darkness for 3 d before being examined using the NightSHADE LB 985 Plant Imaging System (Berthold) as described in Liu et al (2026). For each experimental combination, 3 *N. benthamiana* plants were infiltrated and each leaf was used as one biological replicate.

### Y2H assay

Y2H assays were conducted as described in Fields and Song (1989). The CDSs of *Vv14-3-3A* and *VvDLK* were cloned separately into pGADT7 or into pGBKT7. The CDS of *VvCBF4* was cloned into pGADT7. As negative controls, the respective empty vectors were used. All vectors were transformed into yeast strain AH109 (Takara). Interactions were shown on SC/-Trp/-Leu or SC/-Trp/-Leu/-His medium supplemented with or without 10 mM 3-AT (Biomol GmbH).

### Co-IP assay

Co-IP from agro-infiltrated *N. benthamiana* leaves was conducted according to Muñoz and Castellano (2018) with minor changes as described in Sun et al (2024). Briefly, *35S:Vv14-3-3A-Flag* and *35S:VvDLK-GFP* constructs were introduced into *A. tumefaciens* strain GV3101 and co-infiltrated into leaves of 4-wk-old *N. benthamiana*. Total protein was extracted on ice in homogenization buffer (50 mM Tris-HCl, pH 7.5; 150 mM NaCl; 10 mM MgCl_2_; 0.1% [v/v] Tween 20; 1 mM PMSF; complete protease inhibitor cocktail; Roche) and clarified by centrifugation. Supernatants were incubated overnight at 4 °C with anti-GFP magnetic agarose beads (MBL; D153-11) on an orbital shaker. Beads were collected (500 × g, 2 min, 4 °C), washed, and bound proteins were eluted in 40 μL SDS loading buffer. Immunoblotting was performed using anti-GFP antibody (Proteintech; 1:10,000) and anti-Flag antibody (MBL; M185-11, 1:5,000). Chemiluminescence images were captured with an ultra-sensitive imager (Alliance Q9 Advanced).

### Spinning-disk confocal microscopy

The subcellular localization of VvDLK, also with respect to microtubules, was imaged by spinning-disk confocal microscopy. Tomato seedlings expressing *35S:RFP-VvDLK* either alone or in the background of *35:GFP-AtTuB6* were imaged at day 7 after germination, grown in the light on half-strength MS medium and cold treatment for defined time intervals (see previous). Before imaging, samples were mounted in Milli-Q water, placed on a slide, and covered with a coverslip. The imaging was focusing on the elongating region, in the zone between the hook and the central part of the hypocotyl. For each biological replicate and each time point, ∼10 cells from 3 hypocotyls were imaged and quantified. Suspension cells of *V. rupestris* expressing *35S:GFP-VvDLK* either alone or coexpressing *35S:GFP-VvTuB1/35S:RFP-VvDLK* were imaged during maximal cell expansion at day 5 after subcultivation. Here, aliquots of 30 μL were sampled from the suspension, placed on a slide and covered with a coverslip for microscopy. For each biological replicate and each time point, 15 individual cells were imaged and quantified. For both systems, microtubules were followed in 3 independent experimental series. For quantification of microtubule density, a probing line of 10 µm in length was drawn perpendicular to the orientation of the array and microtubules counted manually (Wang et al 2012). Confocal images were recorded with an AxioObserver Z1 (Zeiss) using a 63× LCI-NeofluarImmCorr DIC objective (NA 1.3) and a spinning-disc device (YOKOGAWA CSU-X1 5,000). GFP was excited at 488 nm, RFP at 561 nm. Confocal z-stacks were captured and processed using the ZEN 2012 (Blue edition) software to generate orthogonal projections from the recorded stacks and to export them in the TIFF format.

### Quantification and statistical analysis

If not specified otherwise, all data in this work were obtained from at least 3 independent experiments with 3 replicates. Values are mean ± SEM. Statistical analyses were carried out in SPSS (version 31.0.1.0, IBM). Paired comparisons were performed using a two-tailed Student's *t* test (*P < 0.05, **P < 0.01). In case of comparisons across more than two samples, a 1-way analysis of variance followed by a Tukey's multiple-comparison test was applied; groups significantly different at *P* < 0.05 are indicated by different lowercase letter. All graphs were generated in GraphPad Prism 10 (GraphPad Software; RRID:SCR_002798). Halftone figures were prepared with Adobe Photoshop CC and BioRender.

## Results

### Overexpression of *VvDLK* reduces cold tolerance

To evaluate the role of VvDLK in the response to cold stress of grapevine cells, we overexpressed a GFP fusion of VvDLK from the genotype *V. vinifera* cv. Pinot Noir PN40024 (the clone that had been used to establish the grapevine reference genome) in suspension cells of *V. rupestris*. Successful transformation was verified using GFP fluorescence (Fig. S1a) and measuring steady-state transcript levels of *VvDLK* (Fig. S1b). Representatively, 3 strains were analyzed, exhibiting moderate (2.5-fold, DLK-OE#4), medium (5-fold, DLK-OE#7), and strong (12-fold, DLK-OE#6) overexpression as compared with the nontransformed wild-type (WT). To assess the effect of *VvDLK* overexpression on cold susceptibility, aliquots of equal fresh weight (0.1 g) from the pooled 3 strains were pregrown, in parallel to the WT, for 7 d at 25 °C and then transferred to 4 °C. After 10 d of this cold treatment, the phenotypes were assessed (Fig. 1a–e). While the growth indices of WT and *VvDLK*-overexpressing lines (DLK-OE) were identical (∼5) if kept at 25 °C, the decrease induced by cold stress was significantly more pronounced in DLK-OE, with a growth index of ∼1.5 as compared with ∼2.5 in the WT. The stronger inhibition of growth was accompanied by elevated oxidative stress (Fig. 1c), evident from stronger signals for superoxide (assayed by NBT chloride) and hydrogen peroxide (assayed by DAB). Likewise, cold-induced mortality was significantly higher in DLK-OE from 48 h after the onset of cold stress (Fig. 1d). As integrative marker for peroxidative damage, we measured MDA contents. While these were identical under 25 °C, they had increased differentially upon cold stress at day 3 after the onset of cold stress, with around 40% higher MDA levels seen in DLK-OE as compared with the WT (Fig. 1e).

**Figure 1:**
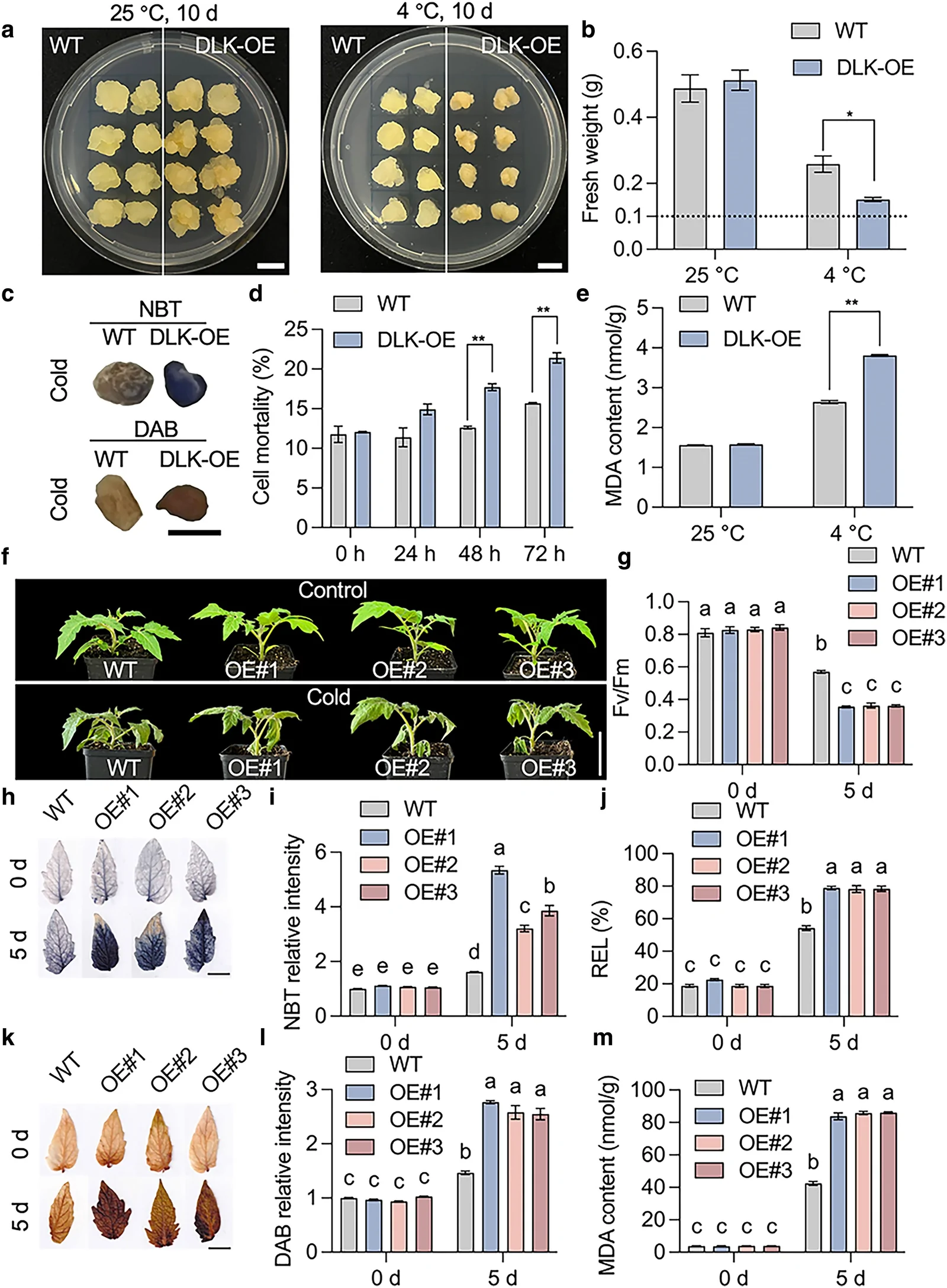
Overexpression of *VvDLK* reduces cold tolerance. (a) Cold stress phenotypes of DLK-OE grapevine calli. The WT and transgenic grapevine calli were grown on medium at 24 °C for 7 d and then treated at 4 °C for another 10 d. Scale bars = 1 cm. (b) Fresh weight of WT and transgenic grapevine calli after cold treatment. Values are mean ± SEM of 3 biological replicates. (c) O_2_•^−^ (Superoxide radicals) and H_2_O_2_ were detected in WT and DLK-OE calli via NBT and DAB staining. Calli following 10 d of cold treatment at 4 °C. Scale bars = 1 cm. (d) Cell death was assessed by Evans blue staining at the indicated time points after cold exposure. Values are mean ± SEM of 3 biological replicates. (e) MDA content of WT and DLK-OE treated with 25 °C or 4 °C. Values are mean ± SEM of 3 biological replicates. (f) Phenotypes of WT and *VvDLK* overexpression (OE#1, OE#2, and OE#3) tomato plants exposed to 25 °C or 4 °C for 5 d. Scale bars = 5 cm. (g) Fv/Fm of WT and OE#1, OE#2, and OE#3 tomato plants exposed to 25 °C or 4 °C for 5 d. (h) NBT and (k) DAB staining of tomato leaves. Scale bars = 1 cm. (i) Quantification of NBT and (l) DAB staining intensity in WT and OE (OE#1, OE#2, and OE#3) after exposure to 25 °C or 4 °C for 5 d. (j) relative electrolyte leakage of WT and OE (OE#1, OE#2, and OE#3) tomato plants exposed to 25 °C or 4 °C for 5 d. (m) MDA content of WT and DLK-OE (OE#1, OE#2, and OE#3) tomato plants exposed to 25 °C or 4 °C for 5 d. Values in panels g, i, j, l, and m are mean ±SEM of 3 biological replicates. Different lowercase letters indicate significant differences (1-way analysis of variance, Tukey's test). Statistical significance was determined by 2-tailed Student's *t* test (*P < 0.05, **P < 0.01).

To investigate the role of VvDLK in planta, an RFP fusion of VvDLK was overexpressed in tomato (cv. Micro-Tom). The lines were verified using the RFP fluorescence and RT-qPCR as readout (Fig. S1c, d). Three lines with low (OE#1) intermediate (OE#3, about twice the expression of OE#1), and strong (OE#2, about 4.5 times the expression of OE#1) were representatively generated in the “Micro-Tom” background. Plants were raised to the 6-leaf stage and then subjected to 4 °C for additional 5 d, when their phenotype was analyzed. The cold-inflicted damage was already visible morphologically by discoloration of the leaves and a loss of turgescence. These symptoms appeared to be more pronounced in the 3 DLK-OE lines (Fig. 1f). To verify this impression, we conducted a couple of physiological assays and observed that the reduction of Fv/Fm (Fig. 1g), the accumulation of superoxide (Fig. 1h, i), the accumulation of hydrogen peroxide (Fig. 1k, l), electrolyte leakage (Fig. 1j), and MDA levels (Fig. 1m) were significantly elevated in the DLK-OE plants as compared with the WT. Collectively, these results demonstrate that overexpression of *VvDLK* increased cold susceptibility. This was already fully manifest even in line OE#1, indicating that even mildly elevated levels are sufficient to cause this phenotype.

Loss-of-function of *SlDLK* improves cold tolerance. The genome of tomato (*Solanum lycopersicum*) encodes a homolog of *VvDLK*, designated *SlDLK*. Motivated by the assumption that the function of this protein is conserved across both species, loss-of-function mutations were generated for *SlDLK* using CRISPR/Cas9. In fact, two lines (CR-2, CR-5) could be identified by the GFP tag inserted in the pYLCRISPR/Cas9 vector and verified by direct sequencing of PCR amplicons spanning the target sites (Fig. 2a, b). When the cold susceptibility of these lines was compared with that of the WT by chilling at 4 °C for 7 d, both lines coped better with reduced symptoms of cold-inflicted damage (Fig. 2c). Again, this was verified using Fv/Fm ratios, accumulation of superoxide and hydrogen peroxide, electrolyte leakage, and MDA levels (Fig. 2d–j). For all of the tested parameters, the mutants performed significantly better than the WT. Thus, while overexpression of *VvDLK* renders the plants more cold-susceptible, the loss of the endogenous *SlDLK* has the opposite effect, which again supports the notion that VvDLK acts as a negative regulator of cold tolerance.

**Figure 2:**
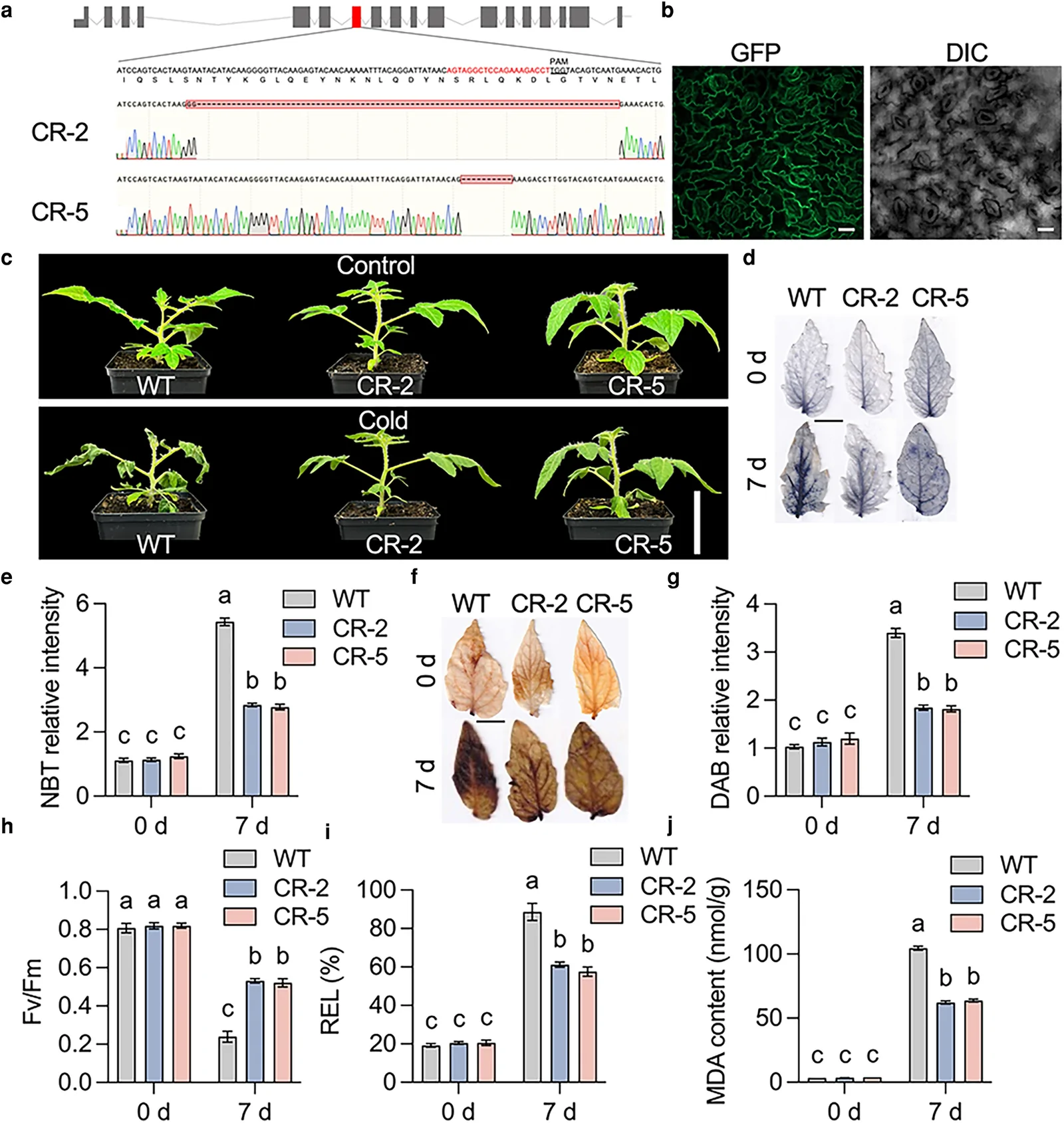
Loss of *SlDLK* function improves cold tolerance. (a) Sequencing results for the knockout of *SlDLK* (CR-2, CR-5 line) target site. (b) The fluorescence detection of Cas9 vector in transgenic tomato leaves. Scale bars = 20 μm. (c) Phenotypes of WT, CR-2, and CR-5 tomato plants exposed to 25 °C or 4 °C for 7 d. Scale bars = 5 cm. (d) NBT and (f) DAB staining of tomato leaves. (e) Quantification of NBT and (g) DAB staining intensity in WT, CR-2, and CR-5 after exposure to 25 °C or 4 °C for 7 d. Scale bars = 1 cm. (h) Fv/Fm of WT, CR-2, and CR-5 tomato plants exposed to 25 °C or 4 °C for 7 d. (i) Relative electrolyte leakage of WT, CR-2, and CR-5 tomato plants exposed to 25 °C or 4 °C for 7 d. (j) MDA content of CR-2 and CR-5 tomato plants exposed to 25 °C or 4 °C for 7 d. Values in panels e, f, g, h, i, and j are mean ±SEM of 3 biological replicates. Different lowercase letters indicate significant differences (1-way analysis of variance, Tukey's test).

### VvDLK accelerates cold-dependent elimination of cortical microtubules

To determine whether VvDLK overexpression influences cold-induced microtubule elimination, we monitored cortical microtubules during cold treatment. To this end, we imaged cells of *V. rupestris* expressing a GFP fusion of grapevine tubulin (VvTuB1) either alone or in combination with an RFP fusion of VvDLK. The cells were treated with low temperature (4 °C) for either 15 or 30 min at day 5 after subcultivation (during the phase of rapid cell expansion (Fig. 3a). In the absence of cold stress, the GFP-tagged tubulin visualized a network of cortical microtubules in both cell lines. The presence of VvDLK did not compromise this network, in the opposite, the microtubules bundles appeared to be thicker as compared with the control. However, already after 15 min at 4 °C, the network in the cells expressing VvDLK seemed more affected than in the control, and this difference became even more accentuated at 30 min. While in the control, still long bundles of microtubules could be detected (albeit their number was lower than in the absence of cold stress), in the overexpressing lines, the GFP tag had distributed through the cytoplasm. The impression that cold-dependent elimination of microtubules is enhanced by overexpression of VvDLK was also confirmed by a quantification of microtubule density (Fig. 3c). The accelerated cold elimination of microtubules upon overexpression of *VvDLK* was also verified in the single *35S:GFP-VvDLK* line visualizing microtubules by immunofluorescence, again backed up by a quantification of microtubule (Fig. S2).

**Figure 3:**
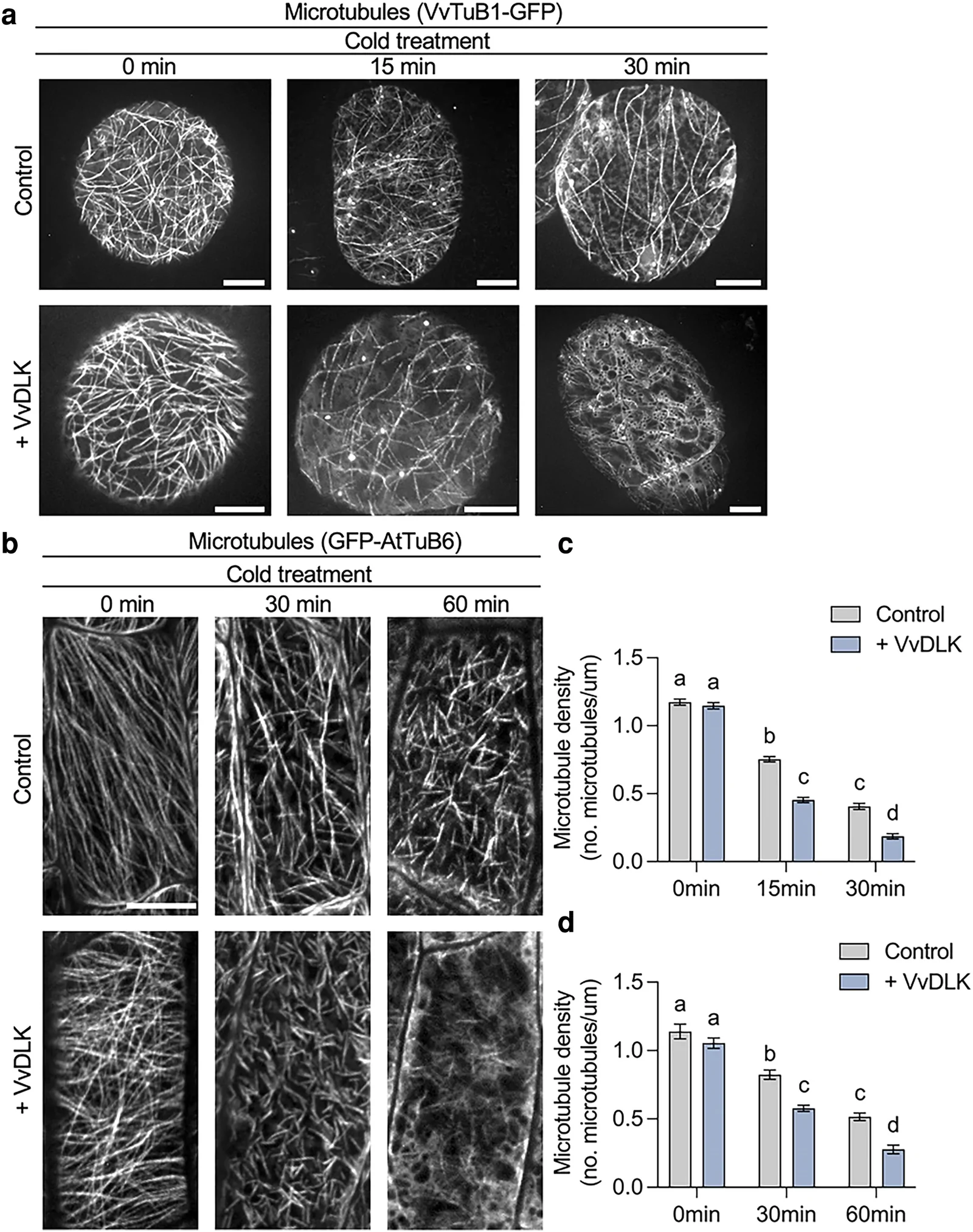
VvDLK promotes cold-induced elimination of cortical microtubules. (a) Representative cortical microtubule networks in the *V. rupestris* suspension cells after cold treatment for 0, 15, and 30 min. Scale bars = 10 μm. (b) Cortical microtubules in light-grown hypocotyls after 0, 30, and 60 min of cold treatment. Scale bars = 10 μm. (c) Quantification of the density of cortical microtubule networks shown in panel a. A total of 15 cells from 3 independent experiments in each condition were analyzed. Values are mean ± SEM. Different lowercase letters indicate significant differences (1-way analysis of variance, Tukey's test). (d) Quantification of the density of cortical microtubule networks shown in panel b. 15 cells from 3 independent hypocotyl cells in each condition were analyzed. Values are mean ± SEM. Different lowercase letters indicate significant differences (1-way analysis of variance, Tukey's test).

To verify, whether this effect of VvDLK exists also in planta, we crossed the tomato lines *35S:GFP-AtTuB6* with 3*5S:RFP-VvDLK* and analyzed the apical region, just basal to the hook. Again, under control conditions, microtubule integrity was not impaired by the overexpression of *VvDLK* under control conditions (Fig. 3b), although a more transverse microtubule orientation indicated that these cells were still not fully elongated. Upon cold stress, the initially long bundles of microtubules were replaced by short, disoriented rods. In the absence of VvDLK, this fragmentation was observed only at 60 min after the onset of cold stress, while the DLK-OE lines exhibited this response already at 30 min. Here, at 60 min, microtubules had vanished with exception of a few residual rods. Instead, a diffuse fluorescence of cytoplasmic strands indicated the presence of tubulin heterodimers. Again, a quantification of microtubule density confirmed that the cold-dependent elimination of microtubules progressed significantly faster in the presence of VvDLK (Fig. 3d). Overall, the earlier and more pronounced elimination of cortical microtubules in DLK-OE grapevine suspension cells and tomato hypocotyl cells (Fig. 3) was associated with increased cold-induced cellular damage and reduced cold tolerance in the physiological assays (Fig. 1). It should be noted that, in contrast to cold-acclimation (Abdrakhamanova et al 2003), this elimination was not transient, but seemed irreversible. This effect was independent of the fact of whether the host species was tomato or grapevine itself and was seen for both cells in suspension and cells that are part of a living tissue.

### Microtubule elimination is not a necessary condition for nuclear import of VvDLK

To examine the subcellular localization of VvDLK in the homologous system, we analyzed grapevine cells expressing GFP-tagged VvDLK by spinning-disc confocal microscopy. This revealed parallel filamentous arrays oriented perpendicular to the long axis of the cells (Fig. 4a), consistent with the characteristic pattern of cortical microtubules. To verify that these filaments were microtubules, we treated the cells with Oryzalin, an herbicide that sequesters dimers from integration into the plus end, such that microtubules are eliminate due to their innate turnover. Oryzalin treatment eliminated the filaments, while the nucleus accumulated the fluorescence (Fig. 4a). In contrast, LatB, targeted against actin filaments, did not destroy the filaments. Also, the solvent control for the two inhibitors (0.1% DMSO) was ineffective. Transient expression of the GFP fusion in epidermal pavement cells of *N. benthamiana* reproduced the same pattern—cortical filaments that were replaced by a nuclear signal upon treatment with oryzalin, while persisting after treatment with LatB or solvent alone (Fig. S3a).

**Figure 4:**
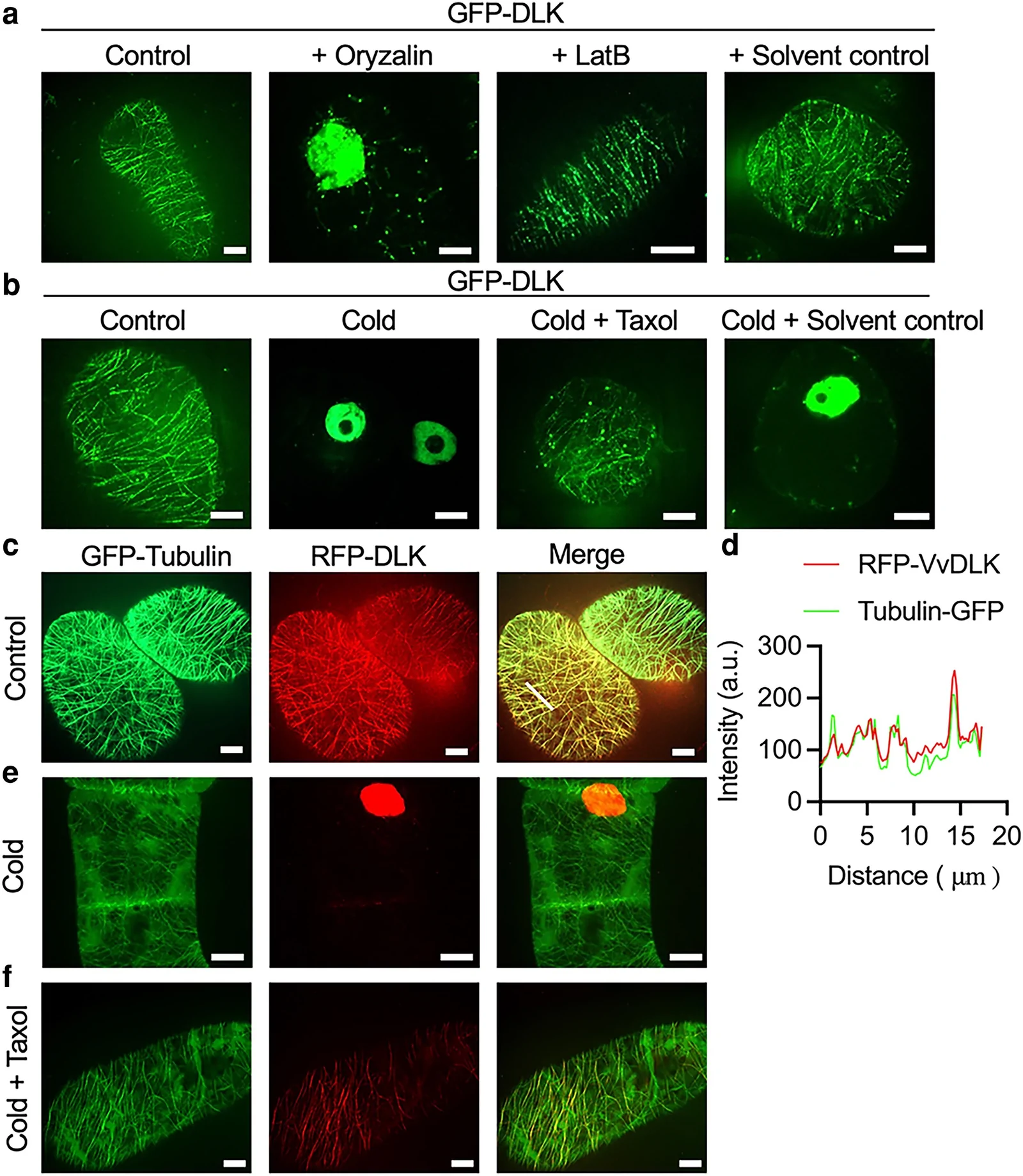
Microtubule elimination is not required for nuclear import of VvDLK. (a) Representative images of GFP-VvDLK under different treatments. Five-day-old transgenic suspension grapevine cells expressing *GFP-VvDLK* were treated with 10 μM oryzalin, 100 nM LatB, and 0.1% DMSO for 30 min. Scale bar = 10 μm. (b) Representative images of GFP-VvDLK under different treatments. Five-day-old transgenic suspension grapevine cells expressing *GFP-VvDLK* were treated with cold, 20 μM Taxol plus cold, and 0.1% DMSO plus cold for 30 min. Scale bars = 10 μm. (c) RFP-VvDLK colocalizes with cortical microtubules detected in stable transgenic grapevine cells. Scale bars = 10 μm. (d) Plot of a line scan drawn in a showing a strong correlation between the spatial localization of RFP-VvDLK and Tubulin-GFP. (e–f) Representative images of suspension grapevine cells coexpressing RFP-VvDLK and Tubulin-GFP under different treatments as described in panel b. Scale bars = 10 μm. a.u., arbitrary units.

To assess whether VvDLK is imported into the nucleus as found for its homologue from rice (Xu et al 2018), we exposed the grapevine cells to cold stress for 30 min. The cortical filaments disappeared completely, while the nucleus became strongly fluorescent (Fig. 4b). When microtubules were stabilized by a pretreatment with taxol, the filaments persisted, albeit partially, and the accumulation in the nucleus was prevented (Fig. 4b).

To test the assumption that the filaments were microtubules, we expressed VvDLK in fusion with RFP in the background of grapevine cells expressing the grapevine b-tubulin VvTuB1 as fusion with GFP. The tight association of both signals (Fig. 4c) also manifest from intensity profiles collected across the filament array (Fig. 4d) supported the notion that VvDLK associates with cortical microtubules.

Because treatments known to eliminate microtubules, such as oryzalin (Fig. 4a) or cold (Fig. 4b), are accompanied by nuclear accumulation of VvDLK, we wondered whether it is the breakdown of microtubules that is responsible for the release of VvDLK that then becomes competent for nuclear import. The simultaneous visualization of VvDLK and microtubules (Fig. 4e) revealed that, upon cold stress, still many microtubules persisted, while simultaneously VvDLK had already completely shifted into the nucleus. Nevertheless, pretreatment with taxol not only stabilized cortical microtubules against cold, but also suppressed nuclear import (Fig. 4f). Again, the same patterns could be confirmed using transient transfection of RFP-VvDLK and GFP-AtTuB6 into *N. benthamiana* (Fig. S3d, e).

Thus, while microtubule elimination seems to be a sufficient condition for nuclear import, it seems not to be necessary, indicating that the association of VvDLK with microtubules is not constitutive and must be regulated. This regulation is linked to microtubule turnover, as it can be intercepted by taxol.

### VvDLK physically interacts with Vv14-3-3a

The pattern seen for VvDLK, negative regulation of cold tolerance and cold-dependent nuclear import, recalls the behavior of 14-3-3 in *A. thaliana* (Catalá et al 2014; Liu et al 2017). This resemblance prompted us to investigate whether specific 14-3-3 proteins interact with VvDLK. Based on a BLAST search and structural modeling with AlphaFold3, we identified two grapevine homologues, which we termed Vv14-3-3A and Vv14-3-3B. We probed these homologues for a potential interaction with VvDLK using BiFC and observed a specific interaction of VvDLK with Vv14-3-3A (Fig. 5c). Interestingly, the reconstituted YFP fluorescence visualized filamentous structures that resembled microtubules. In contrast, we could not detect any interaction between VvDLK and Vv14-3-3B (Fig. S4a). Subcellular localization results showed that Vv14-3-3A itself was located in the cytoplasm (Fig. S5a). The interaction between Vv14-3-3A and VvDLK was further examined using a Y2H assay. All cotransformed yeast strains grew on SC/-Trp/-Leu medium. Transformants coexpressing AD-Vv14-3-3A and BD-VvDLK also grew on SC/-Trp/-Leu/-His medium, whereas negative controls showed no detectable growth (Fig. 5a). To further verify their interaction in planta, we employed the split-luciferase assay following transient expression in leaves of *N. benthamiana* and detected again an interaction between Vv14-3-3A and VvDLK (Fig. 5b). Again, such an interaction was undetectable for Vv14-3-3B (Fig. S4b). To validate this interaction in planta also on the biochemical level, we performed a Co-IP assay using *N. benthamiana* leaves transiently expressing FLAG-Vv14-3-3A together with GFP or GFP-VvDLK. The extracted proteins were incubated with anti-GFP agarose beads, and the bound proteins were detected using an anti-FLAG antibody. In congruence with the data from the BiFC, split-luciferase, and Y2H assays, FLAG-Vv14-3-3A was pulled down by GFP-VvDLK but not by free GFP (Fig. 5d). These results demonstrate the physical interaction between VvDLK and Vv14-3-3A.

**Figure 5:**
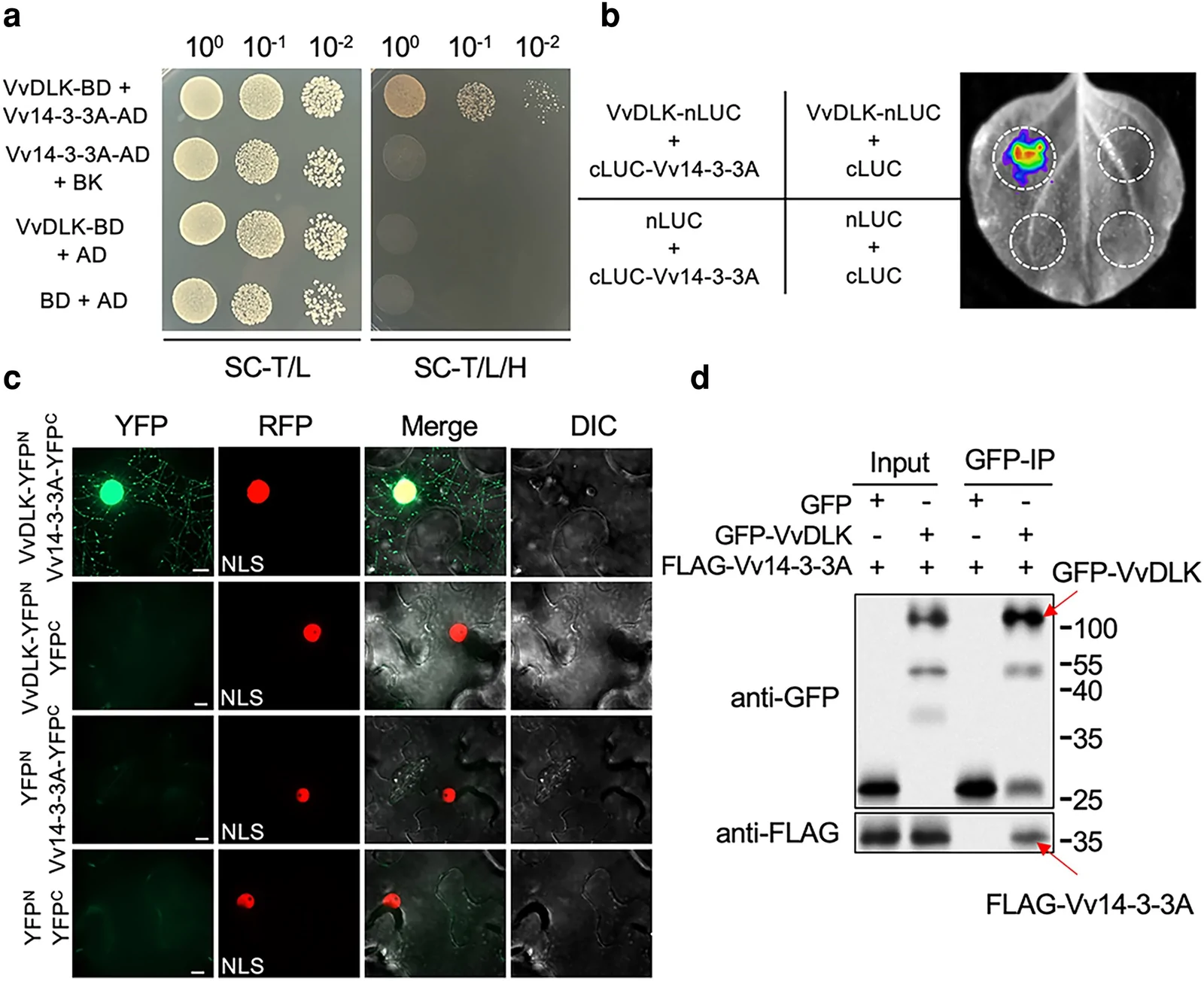
VvDLK interacts with Vv14-3-3A. (a) Y2H assay showing the interaction between VvDLK and Vv14-3-3A. Tenfold serial dilutions (10^0^, 10^−1^, and 10^−2^) of yeast cotransformants were plated on SC/-Trp/-Leu and SC/-Trp/-Leu/-His media. VvDLK-BK + AD, BK + Vv14-3-3A-AD, and BK + AD served as empty vector controls. (b) Split-luciferase (LUC) assay demonstrating the interaction between VvDLK and Vv14-3-3A in *N. benthamiana* leaves. The VvDLK-nLUC + cLUC, nLUC + cLUC-Vv14-3-3A, and nLUC + cLUC were used as negative controls. (c) Bimolecular fluorescence complementation assay showing interaction between VvDLK and Vv14-3-3A in *N. benthamiana* leaves. The controls were VvDLK-YFP^N^ + YFP^C^, YFP^N^ + Vv14-3-3A-YFP^C^, and YFP^N^ + YFP^C^. mCherry-NLS served as a nuclear marker protein. Scale bars = 10 μm. (d) Co-IP assay showing that VvDLK interacts with Vv14-3-3A in *N. benthamiana* leaves.

#### Overexpression of *Vv14-3-3A* reduces cold tolerance

To investigate the contribution of Vv14-3-3A to cold stress responses in grapevine cells, we generated *V. rupestris* suspension cultures overexpressing a GFP-tagged Vv14-3-3A derived from *V. vinifera* cv. Pinot Noir PN40024. To assess the impact of *Vv14-3-3A* overexpression on cold susceptibility, equal fresh weight aliquots (0.1 g) pooled from the three *14-3-3A*–overexpressing (14-3-3A-OE) lines were cultured in parallel with the WT at 25 °C for 7 d and subsequently transferred to 4 °C. After 10 d of cold treatment, phenotypic responses were documented (Fig. 6a–e). At 25 °C, WT and 14-3-3A-OE exhibited comparable growth indices of ∼5. Under cold treatment, however, growth inhibition was markedly stronger in 14-3-3A-OE, whose growth index declined to ∼1.3, compared with ∼2.5 in the WT (Fig. 6b). This enhanced growth suppression was accompanied by increased oxidative stress (Fig. 6c), as indicated by stronger staining for superoxide, detected by NBT, and hydrogen peroxide, detected by DAB. Beginning 48 h after cold treatment, the 14-3-3A-OE cultures exhibited significantly greater cell mortality than the WT. Under 25 °C, MDA levels were comparable between genotypes. By day 3 of cold treatment, however, MDA had increased in both lines, with a substantially stronger rise in 14-3-3A-OE, which accumulated ∼94% more MDA than the WT (Fig. 6e). These results indicate that Vv14-3-3A-OE increases cold susceptibility. To get insight into the functional context of the interaction of VvDLK with Vv14-3-3A, we tested the effect of BV02, a small-molecule antagonist that targets the conserved 14-3-3 binding groove and, thus, interferes with the interaction between 14-3-3 proteins and their clients upon cold-dependent microtubule elimination. Our model was *V. rupestris* cells expressing a GFP fusion of grapevine tubulin β1 in stage of maximal cell expansion (day 5 after subcultivation). While neither the inhibitor nor the solvent (0.1% DMSO) had any effect on microtubule integrity under control conditions (Fig. 6f), we observed that the elimination of microtubules by cold treatment was less pronounced after a preincubation with BV02 (Fig. 6f). A quantification of microtubule integrity revealed that this mitigation was significant (Fig. 6g). These findings suggest that 14-3-3 proteins are involved in cold-induced microtubule elimination. To assess whether Vv14-3-3A is imported into the nucleus, as reported for its *Arabidopsis* homologue (Liu et al 2017), grapevine cells were exposed to cold stress for 6 h. The cytoplasmic signal was partially reduced, while nuclear fluorescence increased markedly (Fig. 6h). The same pattern was confirmed by transient coexpression of GFP-Vv14-3-3A and RFP-NLS in *N. benthamiana* leaves (Fig. S5b).

**Figure 6:**
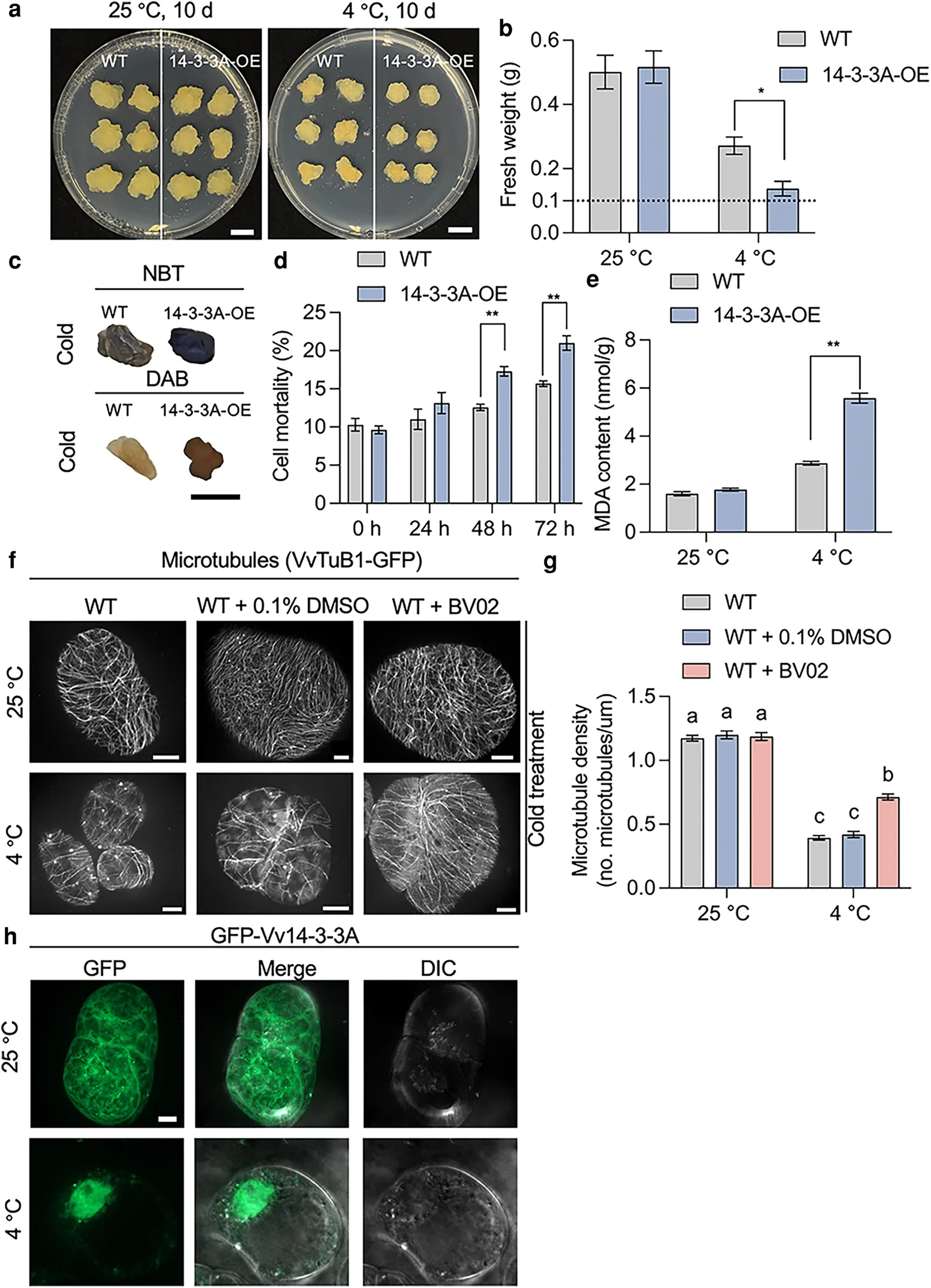
Overexpression of *Vv14-3-3A* reduces cold tolerance. (a) Cold stress phenotypes of 14-3-3A-OE grapevine calli. WT and transgenic grapevine calli were grown on solid medium at 24 °C for 7 d, followed by cold treatment at 4 °C for 10 d. Scale bars = 1 cm. (b) Fresh weight changes in WT and transgenic grapevine calli after exposure to cold stress. Values are mean ± SEM of 3 biological replicates. (c) O_2_•^−^ (superoxide radicals) and H_2_O_2_ were detected in WT and 14-3-3A-OE calli via NBT and DAB staining. Calli following 10 d of cold treatment at 4 °C. Scale bars = 1 cm. (d) Evans blue staining was used to assess cell death at the indicated time points after cold treatment. Values are mean ± SEM of 3 biological replicates. (e) MDA content of WT and 14-3-3A-OE treated with 25 °C or 4 °C. Values are mean ± SEM of 3 biological replicates. (f) Representative images of cortical microtubules *V. rupestris* suspension cells after exposure to 4 °C for 45 min. (g) Quantification of cortical microtubule density. Scale bars = 10 µm. A total of 15 cells from 3 independent experiments in each condition were analyzed. Values are mean ± SEM. Different lowercase letters indicate significant differences (1-way analysis of variance, Tukey's test). (h) GFP-Vv14-3-3A enters the nucleus in response to cold stress. Scale bars = 10 µm. Statistical significance was determined by 2-tailed Student's *t* test (*P < 0.05, **P < 0.01).

### VvDLK and Vv14-3-3a interact with VvCBF4

To determine whether VvCBF4 is associated with the VvDLK–Vv14-3-3A regulatory module, we examined its interactions with both proteins. Subcellular localization analysis showed that VvCBF4 was present in both the cytoplasm and nucleus (Fig. S6a). BiFC assays revealed an interaction between VvCBF4 and VvDLK, with fluorescence signals detected along microtubule-like structures and in the nucleus. By contrast, BiFC signals resulting from the interaction between VvCBF4 and Vv14-3-3A were detected in the cytoplasm and nucleus (Fig. 7a). The interactions of VvCBF4 with VvDLK and Vv14-3-3A were further examined using Y2H assays. Yeast cotransformed with VvCBF4-AD and either VvDLK-BD or Vv14-3-3A-BD grew on both SC/-T/L and SC/-T/L/H media. The corresponding negative controls grew on SC/-T/L medium and showed no detectable growth on SC/-T/L/H medium (Fig. 7b, c). Both interactions were further supported by LCI assays (Fig. 7d, e). To determine whether VvDLK affects VvCBF4 protein accumulation, VvCBF4–Myc was transiently coexpressed with either GFP–Flag as a control or VvDLK–Flag in *N. benthamiana* leaves. To exclude the possibility that reduced VvCBF4 protein accumulation resulted from lower transcript abundance, semi-quantitative RT-PCR was performed. *VvCBF4* transcript levels were similar between the two combinations (Fig. 7f). By contrast, VvCBF4-Myc accumulation was significantly reduced in the presence of VvDLK at 25 °C (Fig. 7f, g). Following 6 h cold treatment, VvCBF4-Myc accumulation remained significantly lower in the presence of VvDLK, although the reduction was less pronounced than at 25 °C (Fig. 7f, g). Together, these results indicate that VvDLK negatively affects VvCBF4 protein accumulation. We further examined whether VvDLK affects the intranuclear distribution of VvCBF4. Under 6 h cold treatment, VvCBF4 predominantly displayed a punctate nuclear distribution, with approximately 96% of cells showing nuclear puncta (Fig. S6a, b). Coexpression of VvDLK reduced this proportion to 72.22% and increased the frequency of cells showing a more homogeneous nuclear distribution. These results indicate that VvDLK alters the intranuclear distribution of VvCBF4 under cold stress.

**Figure 7:**
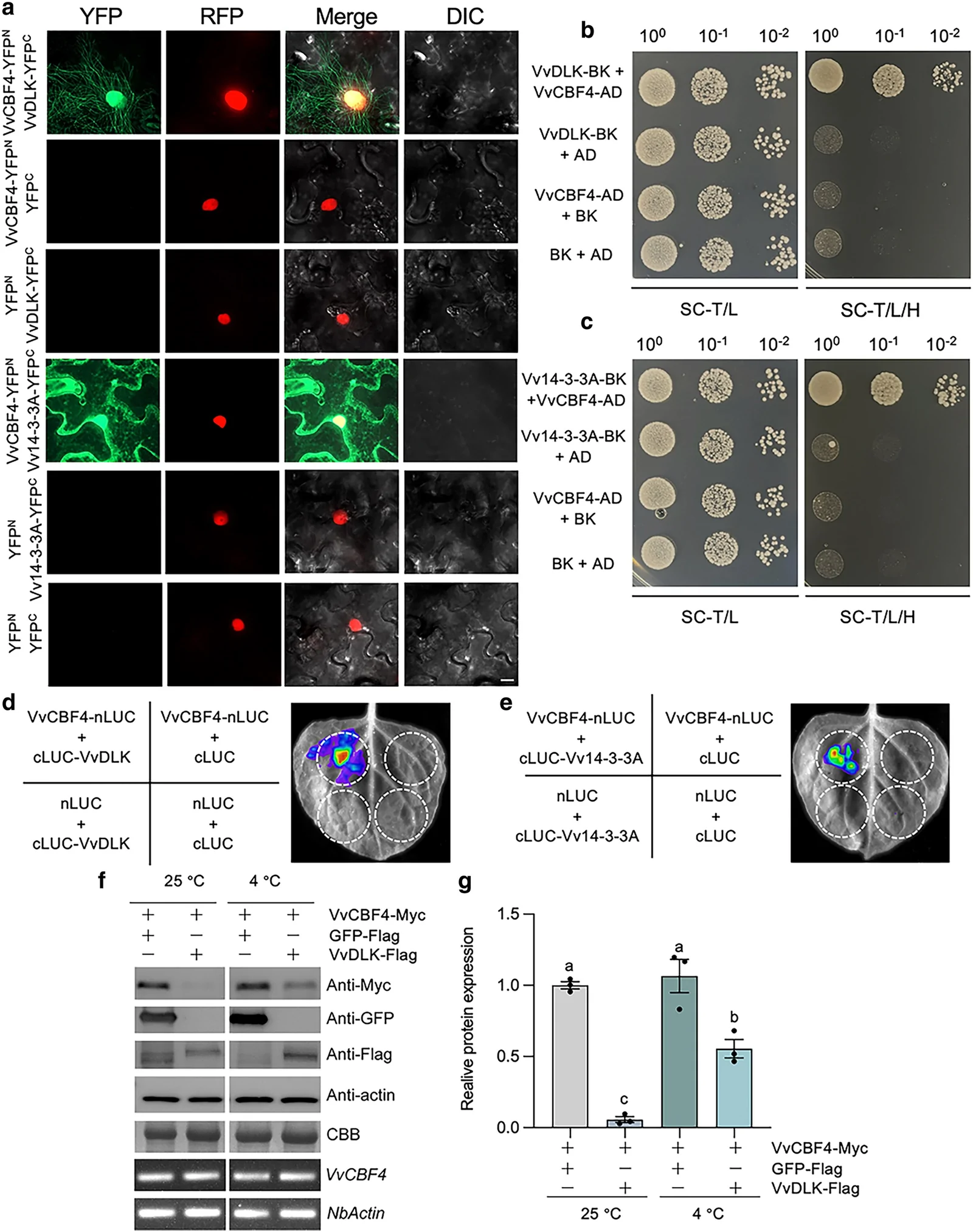
VvDLK and Vv14-3-3A interact with VvCBF4. (a) Bimolecular fluorescence complementation assay showing interaction between VvCBF4 and VvDLK/Vv14-3-3A in *N. benthamiana* leaves. The controls were VvCBF4-YFP^N^ + YFP^C^, YFP^N^ + Vv14-3-3A-YFP^C^, VvDLK-YFP^C^ + YFP^N^, and YFP^N^ + YFP^C^. mCherry-NLS served as a nuclear marker protein. Scale bar = 10 μm. (b) Interaction of VvCBF4 with VvDLK in yeast. Yeast growth is shown on SC/-Trp/-Leu or SC/-Trp/-Leu/-His. (c) Interaction of VvCBF4 with Vv14-3-3A in yeast. Yeast growth is shown on SC/-Trp/-Leu or SC/-Trp/-Leu/-His with 10 mM 3-AT medium. (d) Split-luciferase (LUC) assay demonstrating the interaction between VvDLK and VvCBF4 in *N. benthamiana* leaves. The VvCBF4-nLUC + cLUC, nLUC + cLUC-VvDLK, and nLUC + cLUC were used as negative controls. (e) Split-luciferase (LUC) assay demonstrating the interaction between Vv14-3-3A and VvCBF4 in *N. benthamiana* leaves. The VvCBF4-nLUC + cLUC, nLUC + cLUC-Vv14-3-3A, and nLUC + cLUC were used as negative controls. (f) Immunoblot analysis of VvCBF4-Myc accumulation in *N. benthamiana* leaves transiently coexpressing VvCBF4-Myc with GFP-Flag or VvDLK-Flag under control conditions (25 °C) or after 6 h cold treatment (4 °C). GFP–Flag was used as a negative coexpression control. VvCBF4-Myc, GFP-Flag, and VvDLK-Flag were detected using anti-Myc, anti-GFP, and anti-Flag antibodies, respectively. Actin and Coomassie Brilliant Blue (CBB) staining were used as loading controls. Semi-quantitative RT-PCR was used to examine *VvCBF4* transcript abundance, with *NbActin* as the internal control. (g) Quantification of VvCBF4-Myc protein accumulation shown in panel f. Band intensities were normalized to the corresponding loading control and expressed relative to the 25 °C VvCBF4-Myc + GFP-Flag control. Values are mean ± SEM. Different lowercase letters indicate significant differences (1-way analysis of variance, Tukey's test).

#### *VvDLK* overexpression dampens the cold induction of CBF genes

CBFs are central regulators of cold-induced adaptive gene expression. To determine whether the increased cold susceptibility of DLK-OE cells was associated with an altered CBF response, we compared the transcript levels of 4 CBF genes between nontransformed *V. rupestris* WT and DLK-OE cells after 3 h at 4 °C. Under control conditions, CBF transcript levels did not differ between the two backgrounds. Cold treatment induced all 4 *CBF* genes in both WT and DLK-OE cells, but the magnitude of induction was lower in the DLK-OE cells, particularly for *VrCBF3* and *VrCBF4* (Fig. S7). Thus, elevated VvDLK abundance is associated with a dampened transcriptional induction of *CBF* genes during cold stress.

### VvDLK forms homodimers and dual localization depends on specific domains

Given that most plant kinesins function as dimers to move along the microtubules (Tseng et al 2018), we examined whether VvDLK is capable of self-association. VvDLK dimerization was examined by Y2H, split-luciferase, and BiFC assays. Positive signals were detected only when VvDLK was present in both interaction partners, whereas the corresponding controls were negative (Fig. S8a–c), indicating that VvDLK can form homodimers.

To dissect the roles of the different VvDLK domains for localization, we transiently expressed a panel of VvDLK truncation constructs in *N. benthamiana* leaves (Fig. S9a). Full-length VvDLK displayed the dual localization characteristic for this kinesin with a reticulate, filamentous network spanning the cytoplasm, exhibiting the characteristic features of cortical microtubules along with intracellular localization (Fig. S9b, left) verified by a NLS tagged by RFP (Fig. S9b, center and right). When a GFP-fusion of the N-terminal domain upstream of the coiled-coiled region (VvDLKΔCCMT; aa 1–129) was expressed, no cytoplasmic filaments were to be seen and the signal was confined to the nucleus (Fig. S9c). When the adjacent coiled-coiled domain was included, but leaving out the C-terminal motor domain (VvDLKΔMT; aa 1 to 420), microtubules remained undetectable, but in addition to the nuclear signal, endoplasmic reticulum (ER) strands and lacunae appeared (Fig. S9d). The isolated CC domain (VvDLKCC domain, aa 130–420) did not associate with microtubules, nuclei, or ER but did associate with visualized cytoplasmic speckles (Fig. S9e). In contrast, the two constructs containing the motor domain either including the coiled-coiled region (VvDLKCCMT, aa 130–803) or in isolation (VvDLKMT, aa 421 to 803) visualized microtubules but no nuclear signal (Fig. S9f, g). Interestingly, the signal was almost exclusive to the microtubules, when the motor domain was preceded by the coiled-coiled domain (Fig. S9f), while the motor domain alone was observed to label not only microtubules, but also ER strands (Fig. S9g). Collectively, these data indicate that microtubule binding is mediated by the motor domain, while nuclear localization was mediated by the N-terminal domain upstream of the coiled-coiled domain.

## Discussion

Adaptation to cold stress is crucial for all plants in temperate zones and involves remodeling of cortical microtubules (reviewed in Wang et al 2020), requiring signal-dependent interaction with different MAPs. In the current study, we investigated the role of a DLK, a microtubule motor that can, upon cold stress, move to the nucleus and modulate gene expression (Xu et al 2022). The grapevine homologue of this family turned out to be a negative regulator of cold tolerance, both, in tomato as heterologous system as well as grapevine cells as homologous system. VvDLK was associated with accelerated cold-induced microtubule elimination, underwent rapid nuclear accumulation in response to cold, and physically interacted with Vv14-3-3A. Pharmacological inhibition of 14-3-3 proteins mitigated cold-induced microtubule elimination, suggesting that 14-3-3 proteins participate in this process. In addition, both VvDLK and Vv14-3-3A interact with VvCBF4, linking microtubule remodeling to nuclear cold signaling. These observations raise 3 central issues: How can VvDLK couple microtubule state to nuclear responses? How can VvDLK modulate the microtubule response to cold? What is the functional role of VvDLK for cold acclimation?

### The signal for nuclear import of VvDLK: not presence, but rather turnover

The cold-dependent relocation from microtubules at the plasma membrane into the nucleus is the salient feature of DLK kinesins. The most straightforward scenario for this behavior would be that cold-induced elimination of microtubules would release DLK from its tether, such that it can enter the nucleus and modulate gene expression by virtue of its zinc finger motif in the N-terminal half of the protein (Xu et al 2022). In fact, oryzalin can trigger nuclear import (Fig. 4a), while taxol can suppress this repartitioning of VvDLK (Fig. 4f). The reality is more complex, though: simultaneous labeling of microtubules and VvDLK (Fig. 4e) revealed that relocation of the kinesin can proceed although microtubules still persist. Thus, the seemingly convincing tether hypothesis is wrong. Still, taxol can hinder nuclear import of VvDLK. How can changes in microtubule turnover decide upon release or tethering of a kinesin? The key to this question might be post-translational modifications of tubulin that depend on turnover. In all eukaryotes, a-tubulins carry a C-terminal tyrosine that can be enzymatically cleaved and religated. Detyrosinated tubulin is enriched in stable microtubules, while dynamic microtubules are richer in tyrosinated tubulin. The tubulin tyrosine ligase (TTL) has been identified in porcine brain (Ersfeld et al 1993) and the tyrosine decarboxylase has remained elusive in plants, although a mammalian candidate, vasohibin, has been identified simultaneously by two competing groups (Aillaud et al 2017; Nieuwenhuis et al 2017). There are no plant homologues of vasohibin, though. On the other hand, overexpression of the rice TTL homologue OsTTLL12 promotes tubulin detyrosination (Zhang et al 2021), suggesting that this protein, possibly as part of a complex, can act as tyrosine decarboxylase. This tubulin-modifying enzyme plays an important role in cold sensing, because overexpression of *OsTTLL12* rendered tobacco BY-2 cells more susceptible to cold stress, which was linked with a specific interaction of detyrosinated a-tubulin with phospholipase D, a central signaling hub of cold perception (Zhang et al 2022). One unexpected side effect of overexpression was feedback upon the accumulation of tubulin and TTLL transcripts, which could be modulated by oryzalin and taxol, meaning that microtubule turnover deploys a signal that regulates transcript abundance in the nucleus. A parsimonious explanation of these and the current results would be model, in which cold stress, through phospholipase D, promotes microtubule dynamics and inhibits TTLL12 activity, culminating in lower levels of detyrosinated tubulin, which reduces the affinity of DLK, such that it can be released and translocate to the nucleus. Modulation of kinesin affinity through modulation tubulin detyrosination is not a new phenomenon but was demonstrated earlier for the class-XIV minus-end directed motor KCH (Schneider et al 2015).

### How can VvDLK promote cold-induced microtubule elimination?

We observe that overexpression of VvDLK promotes cold induced microtubule elimination (Fig. 3). The effect of VvDLK on cold-induced microtubule loss could be mitigated by BV02, an inhibitor of 14-3-3 activity (Fig. 6f, g). BV02 inhibits 14-3-3 proteins broadly and therefore cannot assign this effect to a specific isoform (Root et al., 2018). Thus, the BV02 data support the involvement of 14-3-3 proteins in cold-induced microtubule remodeling, while the contribution of individual isoforms remains to be resolved. However, several independent assays demonstrated a physical interaction between VvDLK and Vv14-3-3A (Fig. 5), identifying Vv14-3-3A as a candidate component of this process. Upon overexpression of VvDLK, the pool of VvDLK not associated with 14-3-3A is expected to increase. Through its affinity for stable microtubules, VvDLK would also tether those microtubules to the 14-3-3 scaffold and this is expected to stabilize the cortical cytoskeleton. The 14-3-3 domain is highly conserved throughout the eukaryotes (Camoni et al 2018) and binds to phosphorylated clients, controlling their localization, stability, and interactions (Catalá et al 2014). MAPs that are extensively regulated by phosphorylation especially represent typical interaction partners. In fact, stabilization of microtubules through tethering interacting proteins with the 14-3-3 lattice has been shown earlier for the MAP endosperm defective 1 in *A. thaliana* (Pignocchi and Doonan 2011). Also, kinesins have already been identified as 14-3-3 targets. A prominent example is the kinesin-14 motor Ncd in fruit flies (Repton et al 2022). We hypothesize that excess VvDLK interferes with this tethering and is therefore expected to promote microtubule decay under cold stress, in which the pool of stable microtubules is decreasing anyway. Increasing this pool by taxol should smoothen the competition of 14-3-3 bound and free VvDLK for binding sites at the microtubules and rescue the effect of VvDLK overexpression, which is what we have observed (Fig. 4f). Despite the evolutionary conservation, the interaction of VvDLK is specific, because VvDLK interacts with Vv14-3-3A but not with Vv14-3-3B, in the same experimental framework (Fig. 5; Fig. S4). Overexpression of Vv14-3-3A increases the susceptibility of grapevine cells also (Fig. 6). In cold-induced root growth inhibition, a KOIN–CRPK1 receptor–kinase cascade acts through the 14-3-3–CBF3–SHR pathway, providing an example in which 14-3-3 proteins are embedded in a membrane-to-nucleus cold-signaling module (Zhang et al 2025). In brassinosteroid signaling, 14-3-3 proteins regulate the nucleocytoplasmic distribution of BZR1/OsBZR1 in *Arabidopsis* and rice (*Oryza sativa*) (Bai et al 2007). In salt-stress signaling, Ca^2+^-activated 14-3-3 proteins act as a molecular switch that coordinates SOS2 and PKS5 kinase activities and thereby regulates Na^+^ homeostasis (Yang et al 2019). Thus, we suppose that Vv14-3-3A is a signal transduction component. The molecular mechanisms behind this specificity and signal output are still unknown but could be revealed in the future by mapping 14-3-3 binding motifs on VvDLK and VvCBF4, assessing the phosphorylation dependence of these interactions, and examining how Vv14-3-3A affects VvDLK binding to tyrosinated or detyrosinated microtubules and VvCBF4 protein accumulation.

### Keep cool under cold: VvDLK as an attenuator of cold acclimation

Overexpression of *VvDLK* increases the susceptibility of both grapevine cells and tomato plants to cold (Fig. 1), while disruption of the native tomato homologue by CRISPR-Cas improves cold tolerance (Fig. 2). The elevated cold susceptibility in the DLK-OE grapevine cells is accompanied by a dampened cold induction of all 4 CBF genes, including *CBF4*, the main regulator for cold acclimation (Fig. S7). This pattern is consistent with previous results, in which the rice homologue, *OsDLK*, was overexpressed in tobacco BY-2 cells and the cold induction of *NtCf9* (the tobacco homologue of *CBF4*) was found to be strongly dampened (Xu et al 2022). OsDLK can specifically bind to a motif present in the promoter of this *CBF4* homologue, suggesting a role in modulating its expression. However, *CBF4* is also influenced by the ICE1-dependent transcriptional cascade. In apple, overexpression of the ICE-like transcription factor MdCIbHLH1 increased *MdCBF4* transcript levels (Feng et al 2012). Because DLK acts as a negative regulator, it certainly does not act to convey the primary cold signal through the ICE1–CBF pathway but is rather part of cold habituation, similar to the cold-induced MAP kinase cascades (for review, see Wang et al 2020). In the WT, activation through ICE1-dependent cascade may outweigh negative regulation by endogenous VvDLK, allowing *CBF4* transcripts to accumulate despite the nuclear import of DLK. In the *VvDLK* overexpressors, the elevated abundance of *VvDLK* is expected to strengthen this negative regulation and reduce the amplitude of cold-induced *CBF4* transcript accumulation (Fig. S7). Such restraint is also meaningful, because sustained *CBF* expression can have detrimental effects, including growth retardation (Achard et al 2008). Unlike the other members of the CBF family, CBF4 does not seem to act as element of early cold signaling but is rather activating cold acclimation culminating in frost hardiness (grapevine: Tillett et al 2012; Wang et al 2019; strawberry: Kanbar et al 2024). In addition, it is not constitutively localized in the nucleus as the other CBFs, but imported from the cytoplasm upon cold stress (Shi et al 2022). VvDLK and Vv14-3-3A have been found to interact with VvCBF4 by different assays (Fig. 7), which provides additional evidence that VvDLK may negatively regulate cold tolerance by affecting VvCBF4. Consistent with a post-transcriptional effect, VvDLK reduced VvCBF4 protein accumulation and altered the intranuclear distribution of VvCBF4 under cold stress (Fig. 7f, g; Fig. S6).

DLK-dependent microtubule remodeling may complement the regulation of *Vv*CBF4 expression and VvCBF4 protein accumulation discussed above. Microtubule dynamics can modulate *CBF4* induction during grapevine cold acclimation (Wang et al 2019). VvDLK can also accumulate in the nucleus while cortical microtubules are still present (Fig. 4e), providing access to nuclear interaction partners. The relative contributions of microtubule remodeling and nuclear regulation to the CBF response remain to be established. Through its interactions with VvDLK and VvCBF4, Vv14-3-3A may act as a scaffold that coordinates protein association and subcellular distribution. Such a scaffold function could therefore be integrated into signal transduction. In this model, changes in the association of VvDLK with microtubules would influence its availability to regulate VvCBF4. These regulatory events may operate alongside the classical ICE1–CBF transcriptional cascade and converge on the CBF response. The cold-induced nuclear import of VvCBF4 provides a further level of control over its availability in the nucleus (Shi et al 2022).

Cold stress comes in different colors, either as seasonal effect of advancing fall, inducing CBF-dependent cold acclimation culminating in freezing tolerance by the expression of adaptive genes, a common and necessary event in the life cycle of plants adapted to temperate conditions (for review, see Thomashow 2010). However, plants can also face sudden cold snaps, especially in late spring. Here, the timing does not allow for acclimation and the plant has to rely on rapid mobilization of resources in the older (damaged) leaves that are then shifted to the meristematic tissues that will regenerate new organs once the stress episode is over (for review, see Baena-González 2010). The cold stress in the current study was administered in the form of a cold shock, not in form of an acclimation treatment. Is DLK part of the decision mechanism that quells inappropriate attempts to activate cold acclimation, safeguarding resources for a more appropriate strategy, in which some parts of the plant are sacrificed by cold-dependent programmed cell death to provide resources for the survival of meristematic cells? In fact, congruent with this scenario, the accumulation of CBF4 as well as cold hardening is most efficiently induced in grapevine cells at cool (8 °C) but not cold (0 °C) temperatures (Wang et al 2019). There is a link between cold acclimation and microtubule dynamics (Fig. 8): a rapid but transient elimination of microtubules has been found to be necessary and sufficient to deploy cold acclimation in winter wheat and correlates with elevated levels of tyrosinated tubulin (Abdrakhamanova et al 2003). In response to successful acclimation, microtubules reappear, in a more bundled form, and also with pronounced cold stability. The interactions of microtubules are expected to depend on this change because the pool of detyrosinated tubulin will be limited in naive microtubules and more extensive in experienced, cold-acclimated microtubules. Low temperature induces a transient increase in PLD activity that is dependent on the plant G protein (Liu et al 2024). Detyrosinated tubulin interacts with PLD1a, and when the pool of this tubulin modification is enhanced by overexpression of TTLL12, microtubules acquire resistance against *n*-butanol, a quencher of phosphatidic acids that are normally produced upon G protein–dependent activation of PLD activity (Zhang et al 2022). In naive microtubules, cold stress will increase tyrosination leading to the consequence that the competition of PLDα and DLK for the limited pool of detyrosinated microtubules should become accentuated. This should promote release of the DLK and transfer to the nucleus, thereby dampening cold acclimation. In experienced microtubules, the higher stability will increase the pool of detyrosinated tubulin such that DLK remains tethered and cold-acclimation proceeds unrestrained.

**Figure 8:**
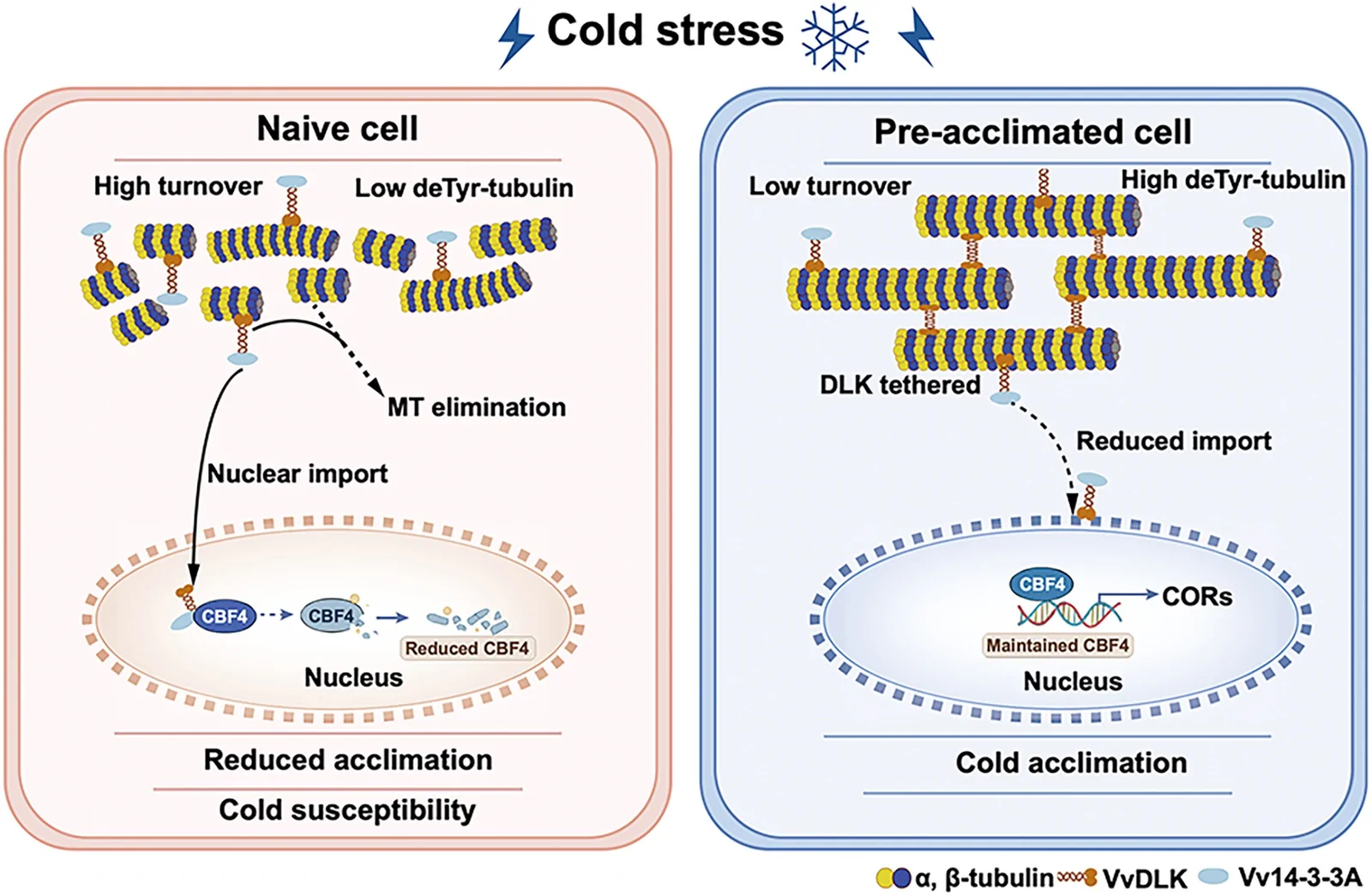
Proposed working model for VvDLK as a microtubule (MT)-dependent attenuator of cold acclimation. In naive cells, cold shock increases cortical MT turnover and promotes VvDLK release from MTs, nuclear import, and attenuation of VvCBF4-dependent cold acclimation, resulting in increased cold susceptibility. Interaction of VvDLK with Vv14-3-3A contributes to cold-induced MT elimination. In preacclimated cells, bundled and more stable cortical MTs with a higher deTyr-tubulin pool retain VvDLK at MTs, reduce its nuclear import, and allow VvCBF4-dependent cold acclimation to proceed.

Therefore, we arrive at a model in which DLK is part of the decision process that a cell challenged with cold has to make: either invest resources in compensating the stress (cold acclimation) or give in and ensure that resources are mobilized and transferred to the younger tissues (cold necrosis). This decision does not only depend on the current stress state of the cell, but also on its previous stress experience (memory). This memory would be reflected as elevated pool of detyrosinated microtubules. The interactions of VvCBF4 with VvDLK and Vv14-3-3A may provide a molecular link between this proposed memory and nuclear cold signaling. Thus, changes in the association of VvDLK would influence its ability to modulate VvCBF4 protein accumulation. In other words, the concurrent interaction of VvDLK with different partners would enable feedback of cold acclimation on cold signaling. Thus, previous stress experience would shape the amplitude and temporal dynamics of the *CBF4* response. We want to emphasize that our experimental setting to administer stress in form of a cold shock will bring certain limitations, paradigmatically illustrating a current debate in plant stress research (for a conceptual opinion, see Plessis 2023): to dissect molecular and cellular mechanisms, plant stress is often investigated in a reductionist setting (as we did in our experiments using the highly artificial setting of a cold shock), while in the real world plants have to cope with combinations of different stresses that arise in variable temporal dynamics. This discrepancy leads to the consequence that the results from laboratory settings show poor transferability to the situation in the field, limiting their usefulness for agricultural application. A crucial factor in this limitation might be the timing of microtubule elimination which is often overlooked in strategies making use of genetic engineering. Enhanced elimination of microtubules due to constitutive overexpression of DLK rendered plants more prone to cold damage. Here, microtubules did not recover. In contrast, when microtubule elimination remains transient, as observed for cold acclimation in winter wheat (Abdrakhamanova et al 2003), this phenomenon heralds cold tolerance. How the transient (as in cold acclimation) or irreversible (as in cold damage) nature of microtubule elimination relates to changes in DLK activity represents a rewarding research question, which could be addressed by comparing DLK localization and activity, as well as microtubule reorganization in naïve plants versus plants prehardened by chilling.

## Supplementary Material

kiag728_Supplementary_Data

## Data Availability

All datasets generated or analyzed during this study are included in this published article and its Supplementary material. The accession numbers of the genes discussed in this article are as follows: *VvDLK* (100254496), *Vv14-3-3A* (100257548), *Vv14-3-3B* (100250345), *VvTuB1* (100244729), *VvCBF1* (AY390372), *VvCBF2* (AY390376), *VvCBF3* (AY390375), *VvCBF4* (100245286), *SlDLK* (101262384), and *AtTuB6* (At5g12250), all of which are available in the National Center for Biotechnology Information database (https://www.ncbi.nlm.nih.gov/).
