# Supplementary material for: VvDLK negatively regulates cold tolerance by interacting with Vv14-3-3A in grapevine
Source: Plant Physiol. 2026 Sep 30;202(2):kiag728. doi: 10.1093/plphys/kiag728 (PMC13688044; doi:10.1093/plphys/kiag728)
Supplement: Supplementary file 1 [file kiag728_supplementary_data.zip › jiang_DLK_ supplementary materials table.pdf]

Table S1 Oligonucleotides used for this study

Construction	Primer	Sequence (5'-3')
	VvDLK-F	GGGGACAAGTTTGTACAAAAAAGCAGGCTTCATGGCTTCCCGTAATCAGAA
	VvDLK-R	GGGGACCACTTTGTACAAGAAAGCTGGGTCTTAGCCATAGCTCAAGCGAG
	VvDLKΔCCMT-F	GGGGACAAGTTTGTACAAAAAAGCAGGCTTCATGGCTTCCCGTAATCAGAACA
	VvDLKΔCCMT-R	GGGGACCACTTTGTACAAGAAAGCTGGGTTTACTGCTCGACAAGTTGGCCT
	VvDLKΔMT-F	GGGGACAAGTTTGTACAAAAAAGCAGGCTTCATGGCTTCCCGTAATCAGAACA
	VvDLKΔMT-R	GGGGACCACTTTGTACAAGAAAGCTGGGTTTATAAATTCTCTCCCTCAATA
	VvDLKCC-F	GGGGACAAGTTTGTACAAAAAAGCAGGCTTCATGGGAAAACTACAGAACGCATTGG
	VvDLKCC-R	GGGGACCACTTTGTACAAGAAAGCTGGGTTTATAAATTCTCTCCCTCAATA
pK7WGF2/ pH7WGR2	VvDLKCCMT-F	GGGGACAAGTTTGTACAAAAAAGCAGGCTTCATGGGAAAACTACAGAACGCATTGG
	VvDLKCCMT-R	GGGGACCACTTTGTACAAGAAAGCTGGGTTTAGCCATAGCTCAAGCGAGAA
	VvDLKMT-F	GGGGACAAGTTTGTACAAAAAAGCAGGCTTCATGCGAAAGAAGCTTCACAACACTA
	VvDLKMT-R	GGGGACCACTTTGTACAAGAAAGCTGGGTTTAGCCATAGCTCAAGCGAGAA
	Vv14-3-3A-F	GGGGACAAGTTTGTACAAAAAAGCAGGCTTCATGGCGGCAGCTCCATCCGCACGCG
	Vv14-3-3A-R	GGGGACCACTTTGTACAAGAAAGCTGGGTTCACTGCTGTTCATCATCACGCTTT
	VvCBF4-F	GGGGACAAGTTTGTACAAAAAAGCAGGCTTCATGAATACTACTTCTCCACCATATTC
	VvCBF4-R	GGGGACCACTTTGTACAAGAAAGCTGGGTCAATAGAGTAACTCCATAACGACA

pK7FWG2	VvTuB1-F	GGGGACAAGTTTGTACAAAAAAGCAGGCTATGAGGGAAATCCTTCATGTTCAA
	VvTuB1-R	GGGGACCACTTTGTACAAGAAAGCTGGGTCCATCGGCTCCAGGCCTTCCTC
RT-PCR	VvCBF4-F	TGCTGGCCTCCACACAC
	VvCBF4-R	CCTGGACGTCTTGTTGGGC
	NbActin-F	GCGGGAAATTGTTAGGGATGT
	NbActin-R	CCATCAGGCAGCTCGTAGCT
	eGFP-F	GTAAACGGCCACAAGTTCA
	eGFP-R	TGTCGGCCATGATATAGACG
	RFP-F	CACGAGTTCGAGATCGAGGG
	RFP-R	TGTCCAGCTTGATGTCGGTC
qPCR	VvEFα-F	GAACTGGGTGCTTGATAGGC
	VvEFα-R	AACCAAAATATCCGGAGTAAAAGA
	SlEFα-F	GGAACTTGAGAAGGAGCCTAAG
	SlEFα-R	CAACACCGACAGCAACAGTCT
	VvDLK-qpcr-F	AGAACAGGCCTCCTCGTAGT
	VvDLK-qpcr-R	CACTTGGAGCCCCCAAATCT
	VrCBF1-qpcr-F	GTGCGGGAACCCCTTAAGAA
	VrCBF1-qpcr-R	TCGGGGAAATTGAGGGAAGC
	VrCBF2-qpcr-F	CCGGTAGATAGTAAGAGCTGGG
	VrCBF2-qpcr-R	GTAAGAAGCAGACCCTCGGC
	VrCBF3-qpcr-F	TTTGCTGCGCTCAATTTCCC
	VrCBF3-qpcr-R	GAGGCGAGTCTTCACTGCAT
	VrCBF4-qpcr-F	AAGAGTTGATGCTGGCCTCC
	VrCBF4-qpcr-R	AAACGTCCCCAGCCATATCC
Split-LUC	VvDLK-nLUC-F	cgagctcggtacccgggatccATGGCTTCCCGTAATCAGAACA
	VvDLK-nLUC-R	cgcgtacgagatctggtcgacGCCATAGCTCAAGCGAGAATCT
	VvDLK-cLUC-F	tacgcgtcccggggcggtaccATGGCTTCCCGTAATCAGAACA
	VvDLK-cLUC-R	atacgaacgaaagctctgcagTTAGCCATAGCTCAAGCGAGAA
	Vv14-3-3A-cLUC-F	tacgcgtcccggggcggtaccATGGCGGCAGCTCCATCC
	Vv14-3-3A-cLUC-R	atacgaacgaaagctctgcagTCACTGCTGTTCATCATCACGC
	Vv14-3-3B-cLUC-F	tacgcgtcccggggcggtaccATGGCTCGCGAGAACTTCGT
	Vv14-3-3B-cLUC-R	atacgaacgaaagctctgcagTCACTCATCGTCTCCACCAGCT
	VvCBF4-nLUC-F	cgagctcggtacccgggatccATGAATACTACTTCTCCACCATATTCCG

	VvCBF4-nLUC-R	cgcgtacgagatctggtcgacAATAGAGTAACTCCATAACGACATGTCA
Y2H	VvDLK-BD-F	atggccatggaggccgaattcATGGCTTCCCGTAATCAGAACA
	VvDLK-BD-R	ccgctgcaggtcgacggatccGCCATAGCTCAAGCGAGAATCT
	VvDLK-AD-F	gccatggaggccagtgaattcATGGCTTCCCGTAATCAGAACA
	VvDLK-AD-R	cagctcgagctcgatggatccGCCATAGCTCAAGCGAGAATCT
	Vv14-3-3A-AD-F	gccatggaggccagtgaattcATGGCGGCAGCTCCATCC
	Vv14-3-3A-AD-R	cagctcgagctcgatggatccCTGCTGTTCATCATCACGCTTT
	Vv14-3-3A-BD-F	atggccatggaggccgaattcGGTTTAGGATTCTATAAATAGAGGCATG
	Vv14-3-3A-BD-R	ccgctgcaggtcgacggatccCTTAATTTCTTGGTAAAGTGTTTTCCTAA
	VvCBF4-AD-F	gccatggaggccagtgaattcATGAATACTACTTCTCCACCATATTCCG
	VvCBF4-AD-R	cagctcgagctcgatggatccCTAAATAGAGTAACTCCATAACGACATGTC
pYLCRISPR/Cas9	Guide RNA	AGTAGGCTCCAGAAAGACCTTGG
	U-F	CTCCGTTTTACCTGTGGAATCG
	gR-R	CGGAGGAAAATTCCATCCAC
	gRT25+	AGTAGGCTCCAGAAAGACCTgttttagagctagaaat
	U6-29T25-	AGGTCTTTCTGGAGCCTACTCaatctcttagtcgact
	SlDLK-F(Seq)	TGCTTATAATAGAGAGAAACATGCC
	SlDLK-R(Seq)	CCATGCCTTCTAACTCGG
BiFC	VvDLK-NE/CE-F	gagaacacgggggactctagaATGGCTTCCCGTAATCAGAACA
	VvDLK-NE/CE-R	gacagtactatcgatggatccGCCATAGCTCAAGCGAGAATCT
	Vv14-3-3A-CE-F	gagaacacgggggactctagaATGGCGGCAGCTCCATCC
	Vv14-3-3A-CE-R	gacagtactatcgatggatccCTGCTGTTCATCATCACGCTTT
	Vv14-3-3B-CE-F	gagaacacgggggactctagaATGGCTCGCGAGAACTTCGT
	Vv14-3-3B-CE-R	gacagtactatcgatggatccCTCATCGTCTCCACCAGCTT
	VvCBF4-NE-F	gagaacacgggggactctagaATGAATACTACTTCTCCACCATATTCCG
	VvCBF4-NE-R	gacagtactatcgatggatccAATAGAGTAACTCCATAACGACATGTCA
Protein	VvDLK-Flag-F	ACAAATCTATCTCTCTGCAGATGGCTTCCCGTAATCAGAA
	VvDLK-Flag-R	TCCTTGTAATCCATGGATCCGCCATAGCTCAAGCGAGAAT
	VvCBF4-Myc-F	TACAAATCTATCTCTCTGCAGGCCACCATGAATACTACTT
	VvCBF4-Myc-F	CAGAAATGAGCTTTTGCTCGGATCCAATAGAGTAACTCCA

Table S2 Medium used for *Vitis rupestris* stable transformation

Medium	Formula
REM medium	MS 4.43 g/L, sucrose 30 g/L, acetosyringone 200 μM/L (pH = 5.8)
COM medium	MS 4.43 g/L, sucrose 30 g/L, 200 mg/L KH_2PO4, 100 mg/L myo-inositol, 1 mg/L thiamine, 0.2 mg/L 2,4-D and 8 g/L phyto agar (pH = 5.8)
SEM medium	MS 4.43 g/L, sucrose 30 g/L, 200 mg/L KH_2PO4, 100 mg/L myo-inositol, 1 mg/L thiamine, 0.2 mg/L 2,4-D, Kan 75 mg/L or Hyg 15 mg/L, timentin 300 mg/L and 8 g/L phyto agar (pH = 5.8)
