# Supplementary material for: VvDLK negatively regulates cold tolerance by interacting with Vv14-3-3A in grapevine
Source: Plant Physiol. 2026 Sep 30;202(2):kiag728. doi: 10.1093/plphys/kiag728 (PMC13688044; doi:10.1093/plphys/kiag728)
Supplement: Supplementary file 1 [file kiag728_supplementary_data.zip › jiang_DLK_ supplementary materials figures .pdf]

Supporting Information

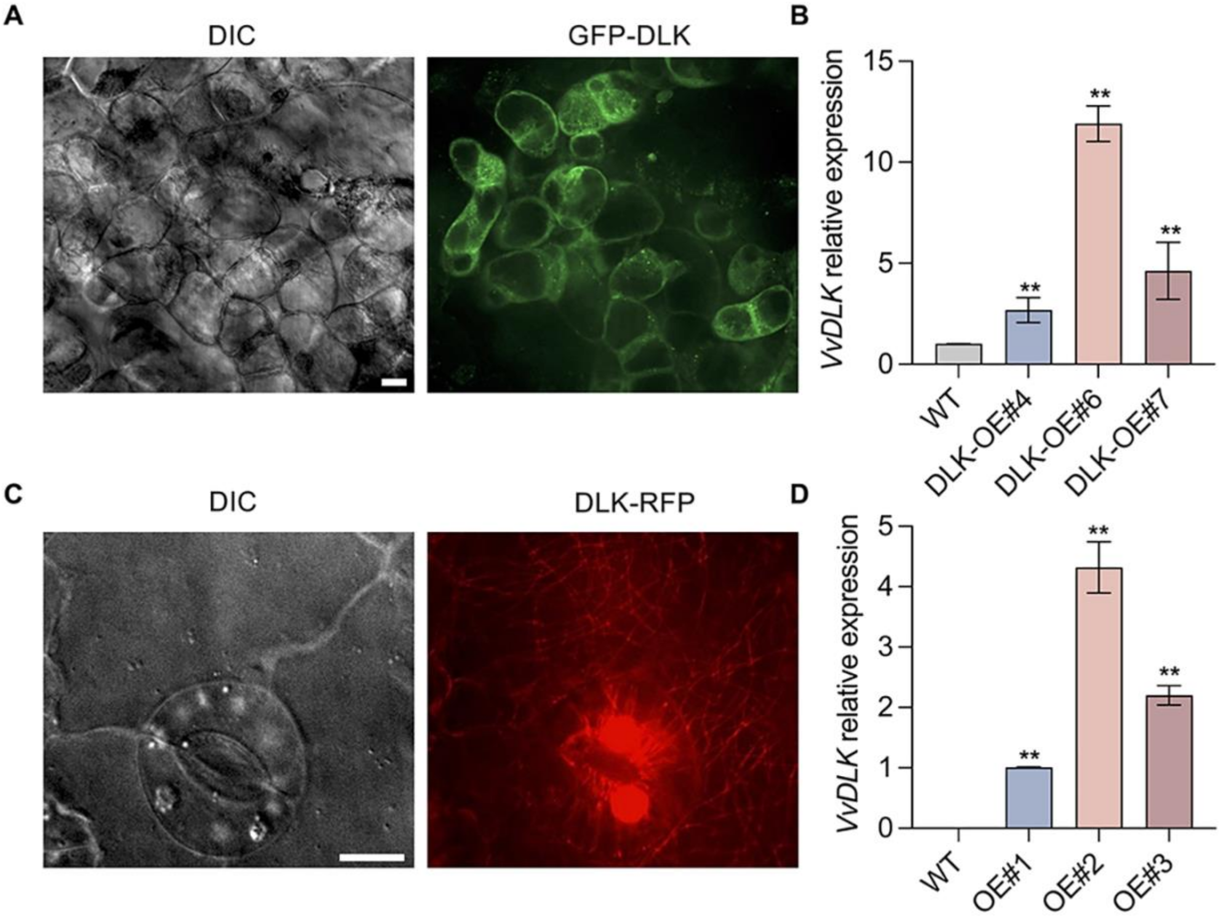

Fig. S1 Identification of stable transformants of grapevine and tomato. (A) Fluorescence detection in transgenic grapevine calli. Scale bars, 20 µm. (B) *VvDLK* relative expression of WT and DLK-OE (#4, #6 and #7). (C) Fluorescence detection in transgenic tomato leaves. Scale bars, 10 µm. (D) *VvDLK* relative expression of WT and DLK-OE (#1, #2 and #3). Values in (B) and (D) are means ± SEM of three biological replicates. Statistical significance was determined by two-tailed Student's t test (*$p < 0.05$. **$p < 0.01$).

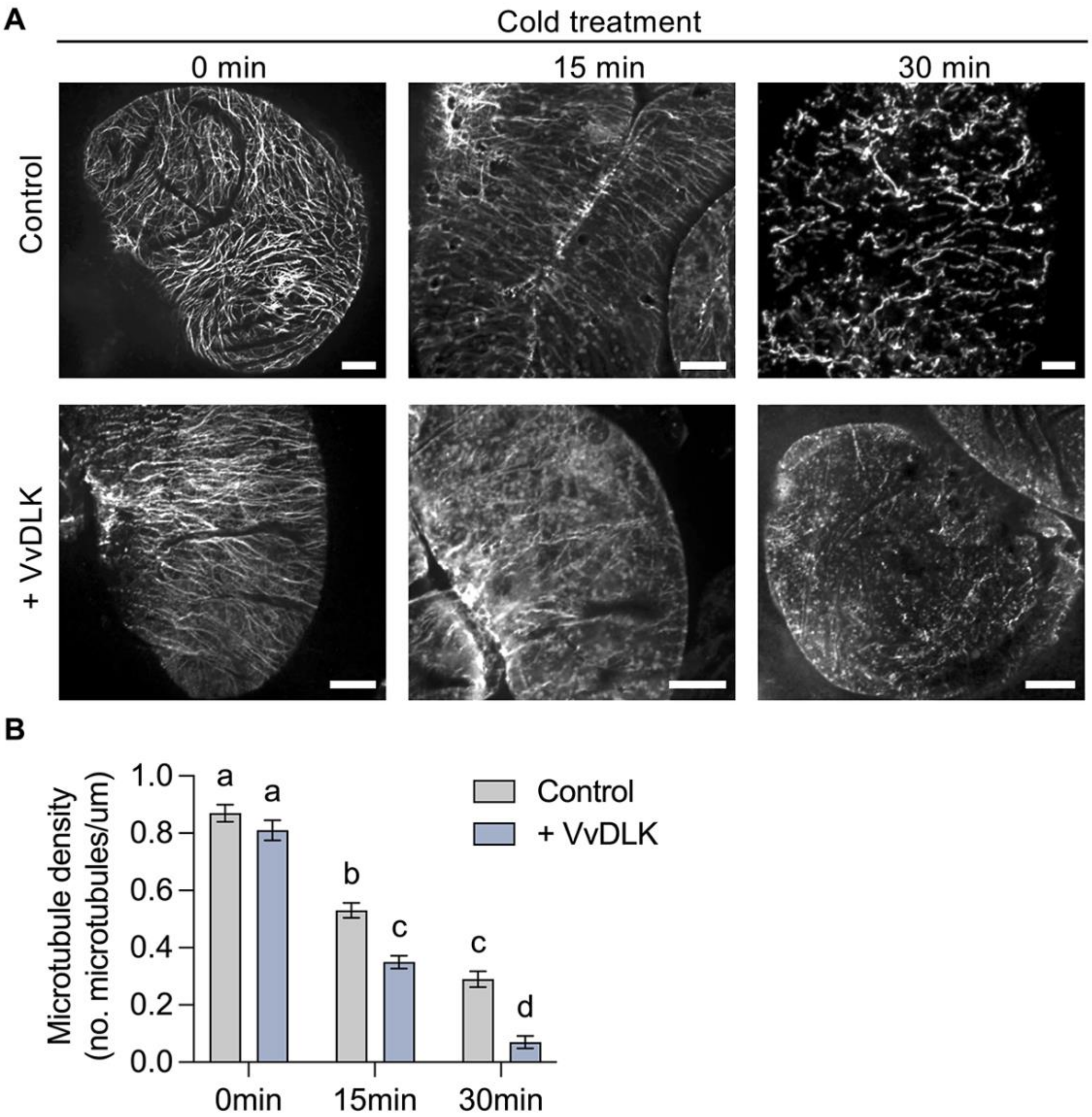

Fig. S2 VvDLK promotes cold-induced cortical microtubule elimination. (A) Representative cortical microtubule networks in *V. rupestris* suspension cells following 0, 15, and 30 min of cold treatment. Microtubules were visualized by immunofluorescence. Scale bar, 10 μm. (B) Quantification of the density of cortical microtubule networks shown in (A). 15 cells from three independent experiments were analyzed for each condition. Data represent means ± SEM of three independent experiments. Different letters indicate significant differences at $P < 0.05$ (One-way ANOVA and Tukey's multiple comparison tests).

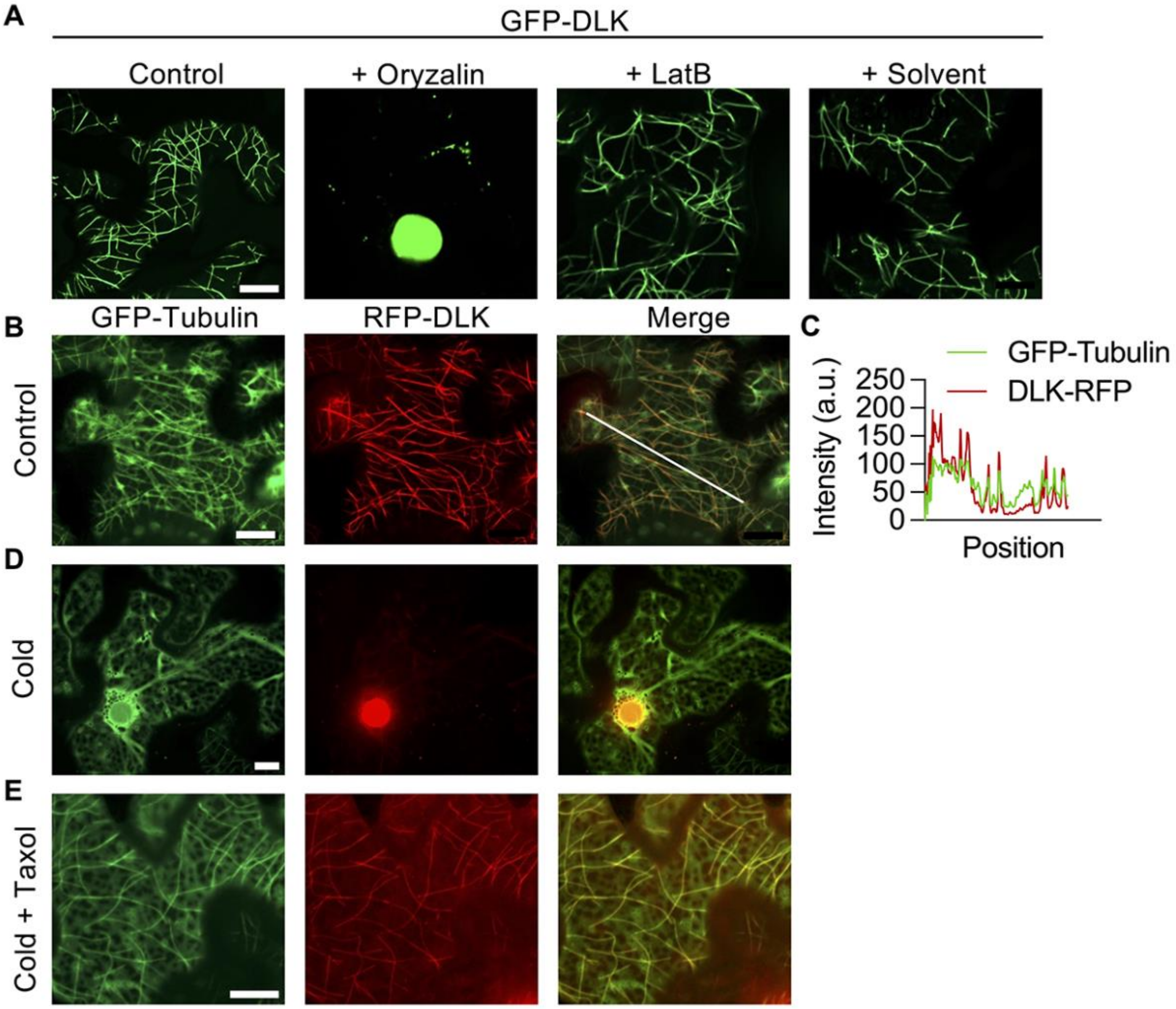

Fig. S3 Nuclear localization of VvDLK in *N. benthamiana* leaves is independent of microtubule elimination. (A) Representative images of GFP-VvDLK under different treatments. *N. benthamiana* leaves transiently expressing GFP-VvDLK were treated with 10 μM oryzalin, 100 nM LatB and 0.1% DMSO for 30 min. Scale bars, 10 μm. (B) RFP-VvDLK colocalizes with cortical microtubules detected in *N. benthamiana* leaves. Scale bars, 10 μm. (C) Plot of a line scan drawn in a showing a strong correlation between the spatial localization of RFP-VvDLK and Tubulin-GFP. (D-E) Representative images of suspension grapevine cells co-expressing RFP-VvDLK and Tubulin-GFP under cold and 20 μM Taxol plus cold treatment for 30 min. Scale bars, 10 μm.

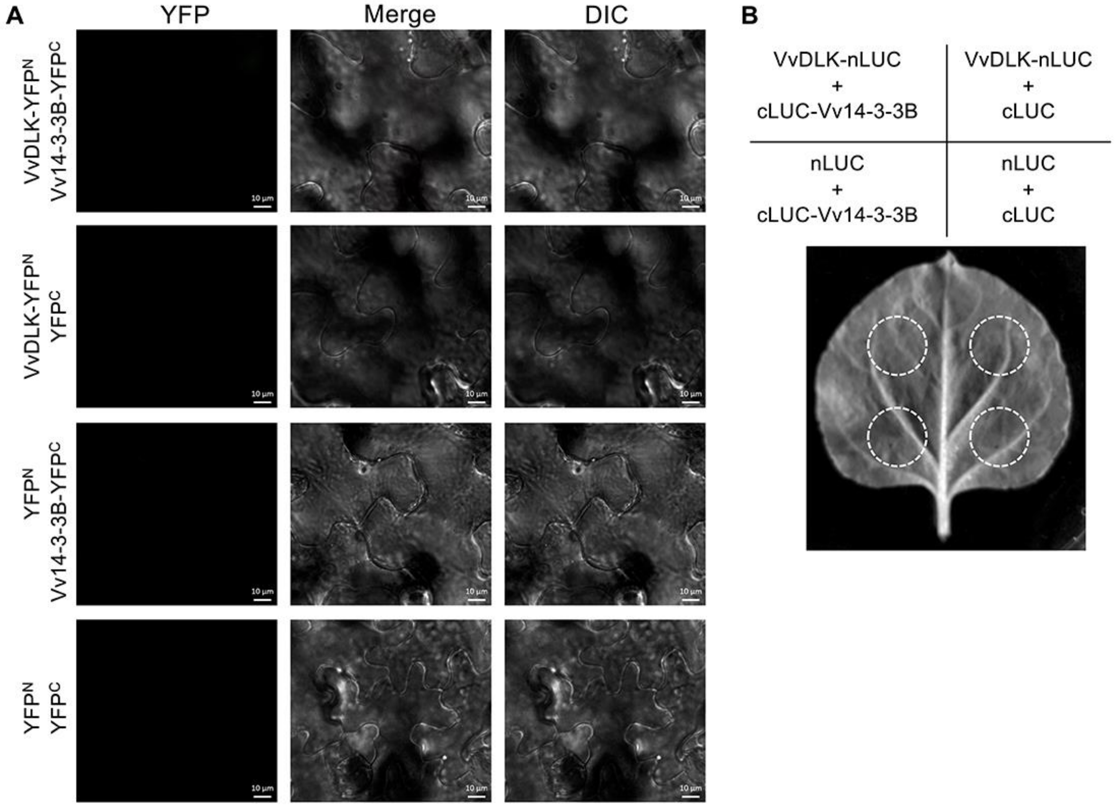

Fig. S4 VvDLK does not interact with Vv14-3-3B. BiFC (A) and LCI (B) assays showed no interaction between VvDLK and Vv14-3-3B.

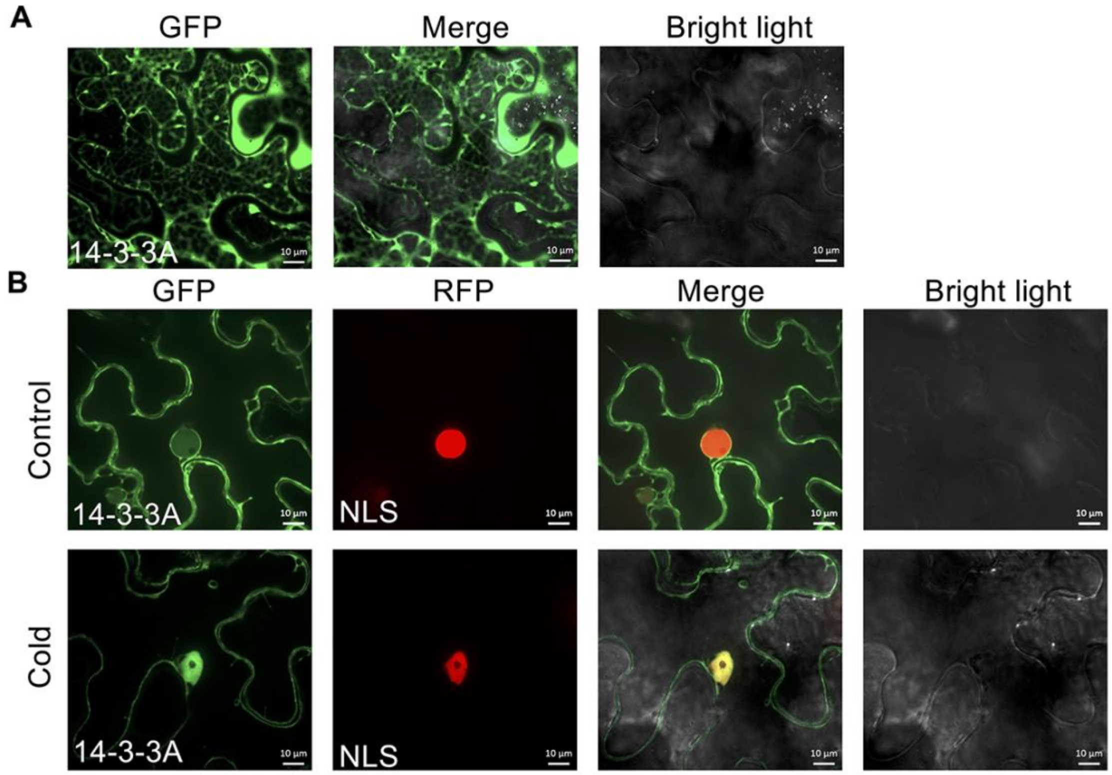

Fig. S5 Subcellular localization of Vv14-3-3A in *N. benthamiana* leaves. (A) Confocal microscopic observation reveals that GFP-Vv14-3-3A localizes to the cytoplasm. (B) Representative images of GFP-Vv14-3-3A under cold treatment. GFP-Vv14-3-3A enters the nucleus in response to cold stress. mCherry-NLS served as a nuclear marker protein. Scale bars, 10 µm.

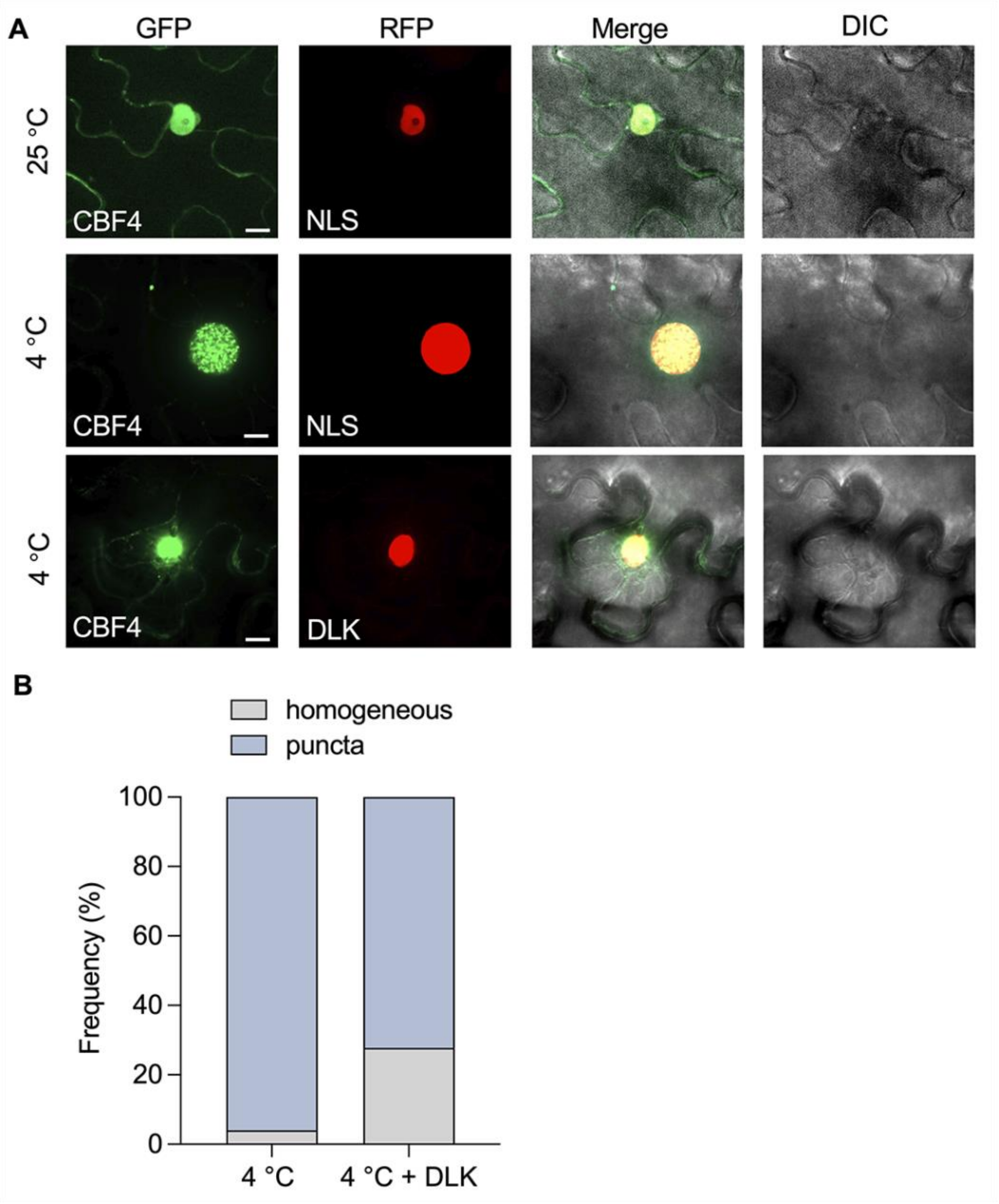

Fig. S6 VvDLK alters the intranuclear distribution of VvCBF4 under cold stress. (A) Subcellular distribution of VvCBF4-GFP in *N. benthamiana* epidermal cells at 25 °C and 4 °C, and following co-expression with VvDLK under cold conditions. RFP-NLS served as a nuclear marker. Scale bars = 10 μm. (B) Percentage of cells showing homogeneous or punctate VvCBF4 nuclear distribution under cold conditions in the absence or presence of VvDLK. For each replicate, cells from from at least 20 randomly selected fields of view were counted (n=3 biological replicates).

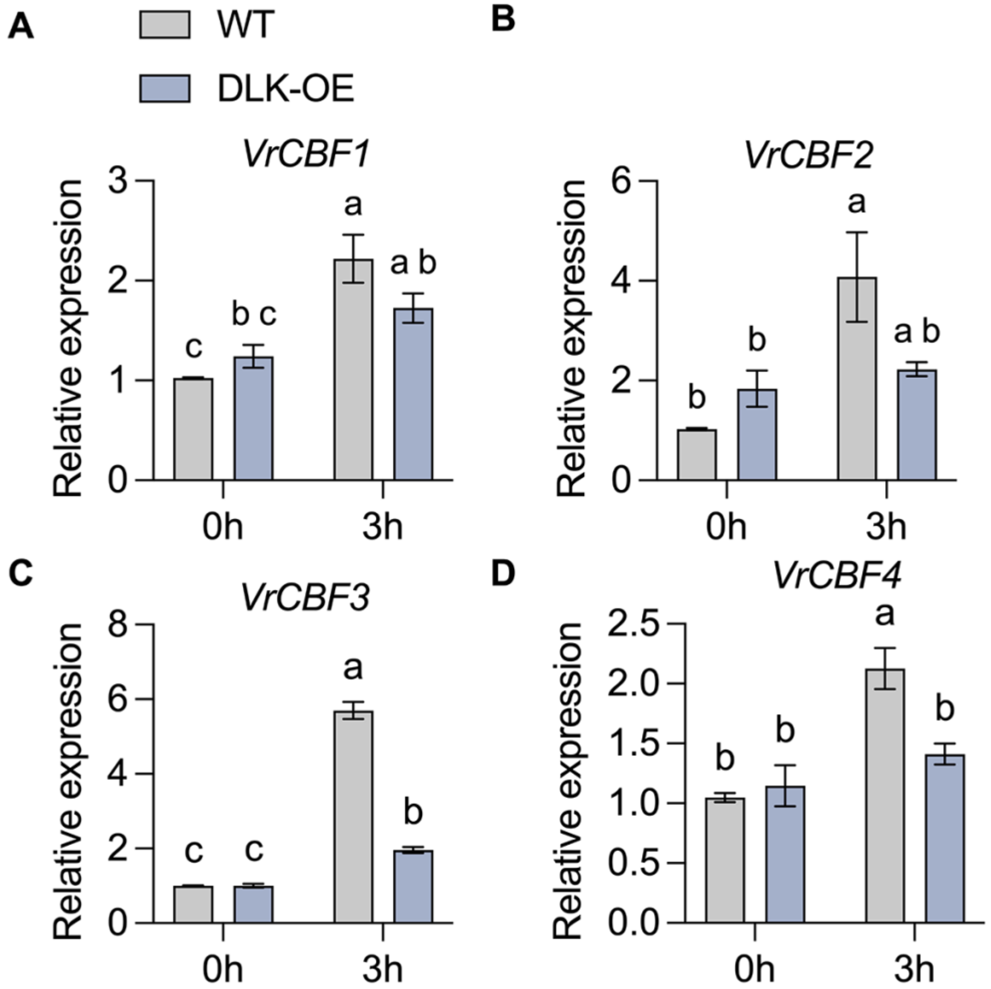

Fig. S7 Relative expression levels of cold-response pathway genes in WT and DLK-OE grapevine cells under cold stress. (A-D) Expression analysis of *VrCBF1*, *VrCBF2*, *VrCBF3* and *VrCBF4* in WT and DLK-OE calli under cold stress based on qRT-PCR. Each reaction was performed in technical triplicates with three independent biological replicates (± SEM). Different letters indicate significant differences at $P < 0.05$ (One-way ANOVA and Tukey's multiple comparison tests).

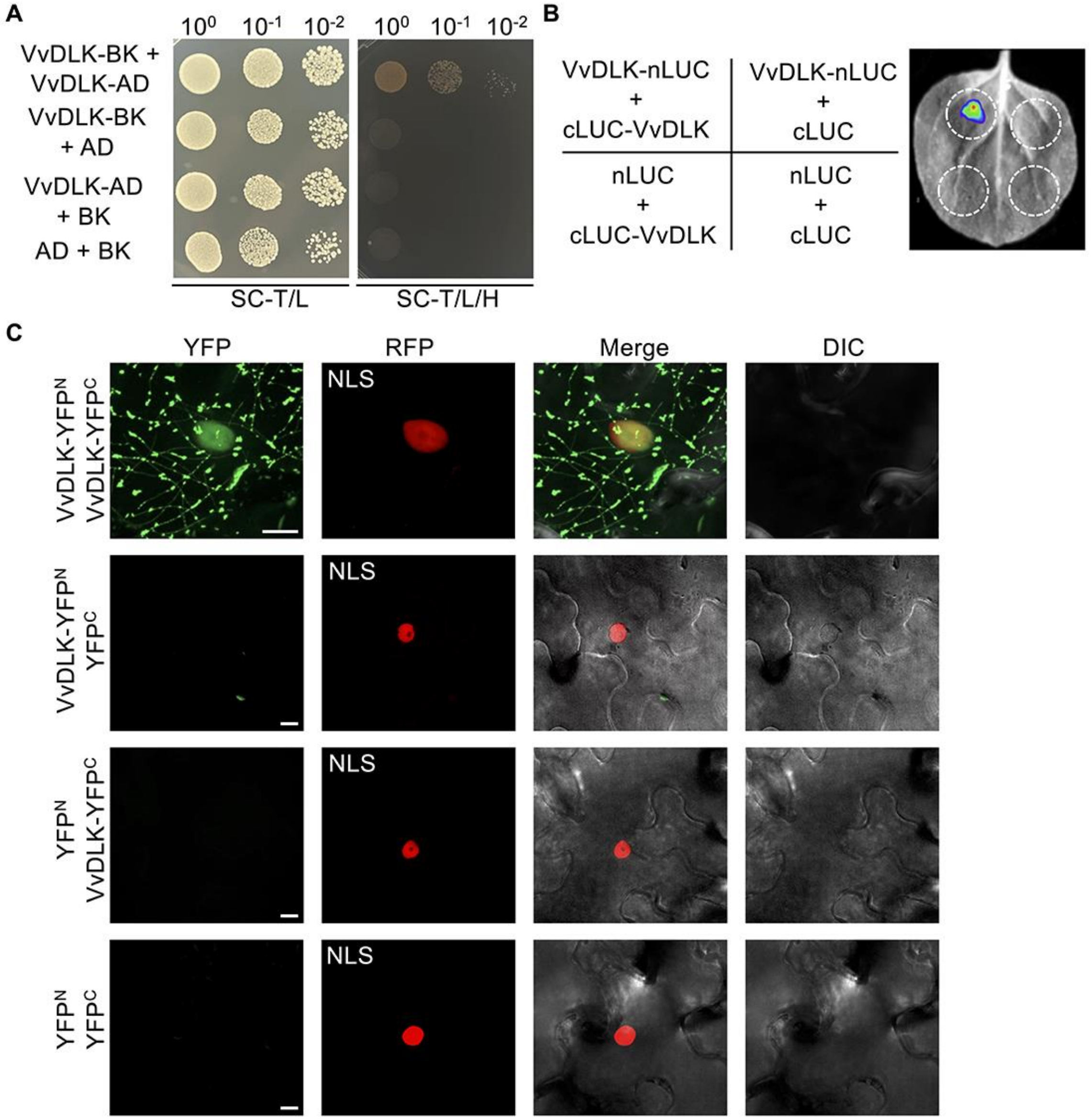

Fig. S8 VvDLK forms a homodimer by protein-protein interaction. (A) VvDLK homo-dimerization tested by protein-protein interaction in yeast. Yeast growth is shown on SC/-Trp/-Leu or SC/-Trp/-Leu/-His. (B) Split-luciferase (LUC) assay demonstrating the VvDLK homo-dimerization in *N. benthamiana* leaves. The VvDLK-nLUC + cLUC, nLUC + cLUC-VvDLK and nLUC + cLUC were used as negative controls. (C) Bimolecular fluorescence complementation assay showing VvDLK homo-dimerization in *N.* benthamiana leaves. The controls were VvDLK-YFP^N + YFP^C, YFP^N + VvDLK-YFP^C and YFP^N + YFP^C. mCherry-NLS served as a nuclear marker protein. Scale bars, 10 μm.

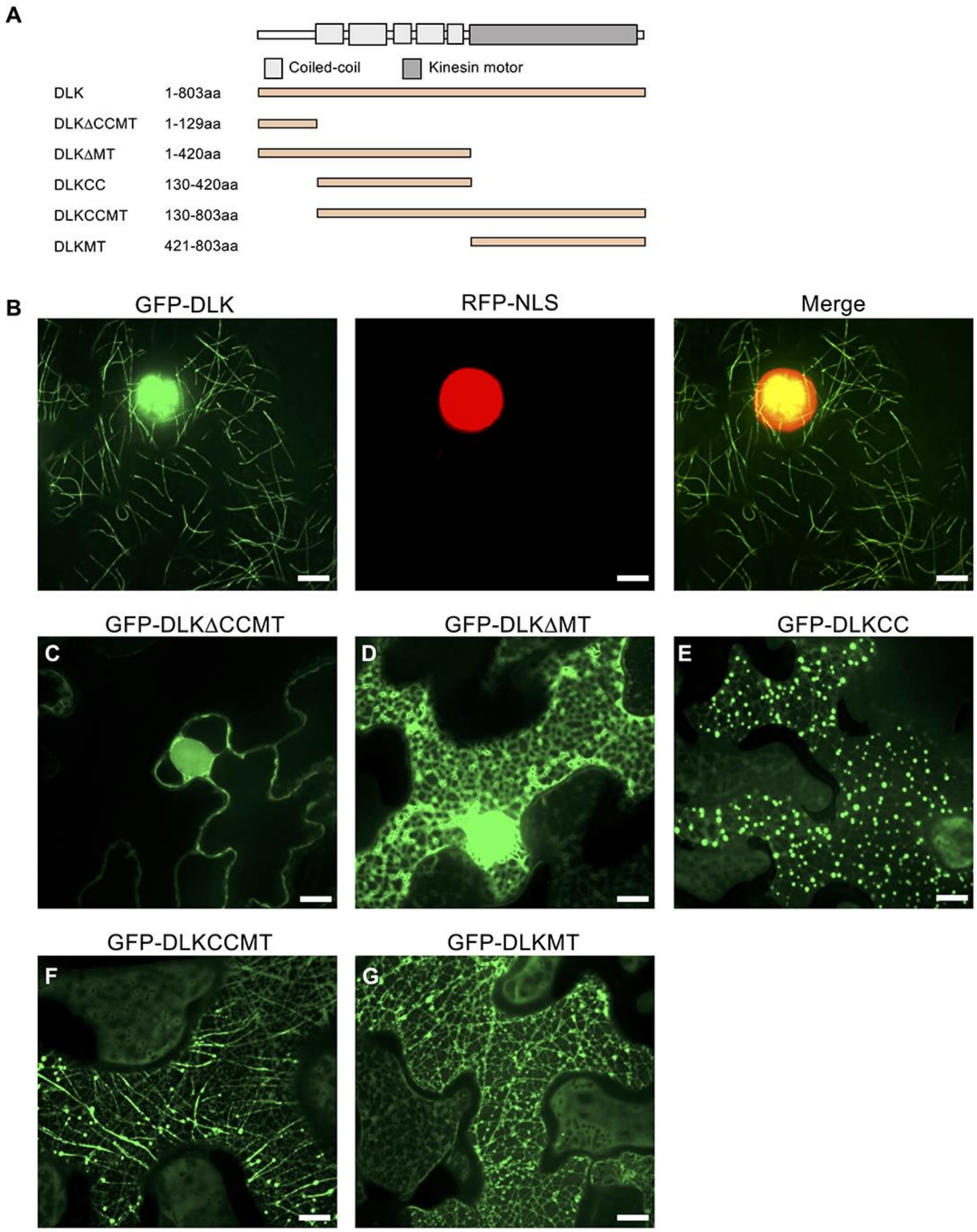

Fig. S9 Subcellular localization of different structural domains of VvDLK in *N. benthamiana* leaves. (A) Schematic diagram of VvDLK functional domains. All truncated proteins were transiently expressed in *N. benthamiana* leaf pavement cells driven by the *35S* promoter. GFP was fused to the N terminus of all proteins. (B) Confocal microscopic observation reveals that GFP-VvDLK localizes to both microtubules and nucleus. mCherry-NLS served as a nuclear marker protein. Scale bar = 10 μm. (C-

D) The GFP-VvDLKΔCCMT and GFP-VvDLKΔMT were not localized to microtubules but were observed in the nucleus. Scale bar = 10 μm. (E) The GFP-VvDLKCC failed to localize to either microtubules or the nucleus. Scale bar = 10 μm. (F-G) The GFP-VvDLKCCMT, GFP-VvDLKMT showed microtubule localization. Scale bar = 10 μm.
